# The prevalence, correlation, and co-occurrence of neuropathology in old age: harmonisation of 12 measures across six community-based autopsy studies of dementia

**DOI:** 10.1016/S2666-7568(23)00019-3

**Published:** 2023-03

**Authors:** Emma Nichols, Richard Merrick, Simon I Hay, Dibya Himali, Jayandra J Himali, Sally Hunter, Hannah A D Keage, Caitlin S Latimer, Matthew R Scott, Jaimie D Steinmetz, Jamie M Walker, Stephen B Wharton, Crystal D Wiedner, Paul K Crane, C Dirk Keene, Lenore J Launer, Fiona E Matthews, Julie Schneider, Sudha Seshadri, Lon White, Carol Brayne, Theo Vos

**Affiliations:** a Institute for Health Metrics and Evaluation, University of Washington, Seattle, WA, USA; b Cambridge Public Health, University of Cambridge, Cambridge, UK; c Framingham Heart Study, Framingham, MA, USA; d Glenn Biggs Institute for Alzheimer's & Neurodegenerative Diseases, UT Health San Antonio, San Antonio, TX, USA; e Department of Neurology, Boston University School of Medicine, Boston, MA, USA; f Department of Biostatistics, Boston University School of Public Health, Boston, MA, USA; g Cognitive Ageing and Impairment Neurosciences Lab, Justice and Society, University of South Australia, Adelaide, SA, Australia; h Department of Laboratory Medicine and Pathology, University of Washington School of Medicine, Seattle, WA, USA; i Department of Pathology, Molecular and Cell Based Medicine, Icahn School of Medicine at Mt Sinai, New York, NY, USA; j Sheffield Institute for Translational Neuroscience, University of Sheffield, Sheffield, UK; k Department of Medicine, University of Washington, Seattle, WA, USA; l Laboratory of Epidemiology and Population Sciences, Intramural Research Program, National Institute on Aging, Baltimore, MD, USA; m Population Health Sciences Institute, Newcastle University, Newcastle upon Tyne, UK; n Rush Alzheimer's Disease Center, Chicago, IL, USA; o Rush University Medical Center, Chicago, IL, USA; p Pacific Health Research and Education Institute, Honolulu, HI, USA

## Abstract

**Background:**

Population-based autopsy studies provide valuable insights into the causes of dementia but are limited by sample size and restriction to specific populations. Harmonisation across studies increases statistical power and allows meaningful comparisons between studies. We aimed to harmonise neuropathology measures across studies and assess the prevalence, correlation, and co-occurrence of neuropathologies in the ageing population.

**Methods:**

We combined data from six community-based autopsy cohorts in the US and the UK in a coordinated cross-sectional analysis. Among all decedents aged 80 years or older, we assessed 12 neuropathologies known to be associated with dementia: arteriolosclerosis, atherosclerosis, macroinfarcts, microinfarcts, lacunes, cerebral amyloid angiopathy, Braak neurofibrillary tangle stage, Consortium to Establish a Registry for Alzheimer's disease (CERAD) diffuse plaque score, CERAD neuritic plaque score, hippocampal sclerosis, limbic-predominant age-related TDP-43 encephalopathy neuropathologic change (LATE-NC), and Lewy body pathology. We divided measures into three groups describing level of confidence (low, moderate, and high) in harmonisation. We described the prevalence, correlations, and co-occurrence of neuropathologies.

**Findings:**

The cohorts included 4354 decedents aged 80 years or older with autopsy data. All cohorts included more women than men, with the exception of one study that only included men, and all cohorts included decedents at older ages (range of mean age at death across cohorts 88·0–91·6 years). Measures of Alzheimer's disease neuropathological change, Braak stage and CERAD scores, were in the high confidence category, whereas measures of vascular neuropathologies were in the low (arterioloscerosis, atherosclerosis, cerebral amyloid angiopathy, and lacunes) or moderate (macroinfarcts and microinfarcts) categories. Neuropathology prevalence and co-occurrence was high (2443 [91%] of 2695 participants had more than one of six key neuropathologies and 1106 [41%] of 2695 had three or more). Co-occurrence was strongly but not deterministically associated with dementia status. Vascular and Alzheimer's disease features clustered separately in correlation analyses, and LATE-NC had moderate associations with Alzheimer's disease measures (eg, Braak stage ρ=0·31 [95% CI 0·20–0·42]).

**Interpretation:**

Higher variability and more inconsistency in the measurement of vascular neuropathologies compared with the measurement of Alzheimer's disease neuropathological change suggests the development of new frameworks for the measurement of vascular neuropathologies might be helpful. Results highlight the complexity and multi-morbidity of the brain pathologies that underlie dementia in older adults and suggest that prevention efforts and treatments should be multifaceted.

**Funding:**

Gates Ventures.

## Introduction

The number of individuals living with dementia globally is estimated to be more than 55 million, and this number is expected to increase to over 150 million by 2050.^1^ Despite the large and increasing magnitude of the global burden of dementia, many outstanding questions remain regarding the underlying causes that lead to symptomatic disease.^2^ The amyloid cascade hypothesis was first proposed over 30 years ago to explain the biological underpinnings of Alzheimer's disease and substantial funding has been directed towards basic research and drug development for Alzheimer's disease in years since.^3^ However, recent evidence and largely null effects on cognition from clinical trials of amyloid immunotherapies have led to more questions than answers.^4^ There have been consistent calls to diversify research beyond these hypotheses as epidemiological research in vivo and after death have indicated greater diversity of underlying mechanisms.^5^ Deeper understanding of the changes in the ageing brain is clearly needed to untangle more of the causes of late-life dementia.

**Research in context**

**Evidence before this study**

We searched PubMed for studies published in English from Jan 1, 1980, until Aug 15, 2022, using the key terms “neuropath*”, “dementia”, and “autopsy”. Most available studies have been done in patients derived from clinical series and settings. Few population-based and community-based cohorts exist. Those that do exist usually either present one cohort, or combine different cohorts from within the same centre. One publication combined two studies and two papers combined two or three population-based studies in Europe to increase power to examine education and neglected pathologies. A fourth focused on those without cognitive impairment across several studies, with a further large exercise focused on one neuropathology (TDP-43 and limbic-predominant age-related TDP-43 encephalopathy neuropathologic change [LATE NC]). None of the identified studies combined data on multiple pathologies in participants across the spectrum of cognitive impairment using more than two or three community-based and population-based autopsy studies.

**Added value of this study**

Through our harmonisation of six community-based autopsy studies and incorporation of new findings on LATE-NC, we provide compelling evidence on the co-occurrence of neuropathologies as well as their inter-relationships. Although such evidence has been reported from single or, at the most, two to three studies, bringing these six studies together shows the importance of multiple pathologies and their inter-relationships in a way that should help direct future research into the underpinnings of dementia in older populations. We also highlight challenges to harmonisation of neuropathology cohorts and emphasise inconsistencies in the measurement of vascular neuropathologies.

**Implications of all the available evidence**

This study highlights the need for consistent approaches to the measurement of vascular pathologies in the brain and shows that efforts in the 1980s to create more standardised methods for the measurement of Alzheimer's disease-type pathologies have been useful. Our findings indicate high neuropathological burden along with high co-occurrence of neuropathologies, including Alzheimer's disease and vascular pathologies, and emphasise the importance of considering and measuring various neuropathologies in future research. With improvements to the standardisation of non-Alzheimer's disease pathologies, harmonised datasets of population-based and community-based autopsy studies have the potential to lend new insights to the causes of neuropathologic burden and clinical dementia.

Autopsy studies can provide important insights into biological processes in the brain because they allow for detailed characterisation of a wider range of neuropathologies with greater precision than is possible with less precise in vivo measures, such as amyloid and tau PET scans or biofluid biomarkers. However, most autopsy studies are done in selected clinical samples, which can lead to bias in the characterisation of neuropathologies when there are factors associated with both neuropathology prevalence and inclusion in the study. Similarly, studies on associations between risk factors and a neuropathological outcome could be biased if a factor were associated with selection, as well as with the exposure and outcome of interest. For example, in the Adult Changes in Thought (ACT) study, associations between dementia and neuropathologies were significantly different when comparing those with clinic visits versus when using all data (representative sample) for four of eight neuropathologies considered.^6^

Although population-based autopsy studies can lead to novel findings, many are limited by small sample size, or restrictions to specific geographical areas or subpopulations. Bringing studies together enables comparison of results and the possibility of pooling data. However, differences in measurement procedures across studies must be explored before pooling is possible. Previous efforts to harmonise population-based autopsy studies have either focused on specific questions about the association between a risk factor and pathology,^7^ have only included a few cohorts,^8^ or have focused on a single neuropathology.^9^ To our knowledge, no work to date has sought to characterise the prevalence of neuropathologies in the general population across more than two or three population-based neuropathology cohorts.

We aimed to first assess the feasibility of harmonising data across a set of population-based autopsy studies and then to describe the burden of neuropathologies in the general ageing population. We used data from six community-based studies and compared measurement procedures and prevalence of neuropathologies. We also examined correlations between the neuropathologies considered and the co-occurrence of key neuropathologies.

## Methods

### Data sources

We used data from six autopsy cohorts: the ACT study, the Framingham Heart Study (FHS), the Cambridge City Over-75s Cohort Study (CC75C), the Cognitive Function and Ageing Studies (CFAS), the Honolulu-Asia Aging Study (HAAS), and the Religious Orders Study and Memory and Aging Project (ROSMAP).^10^, ^11^, ^12^, ^13^, ^14^, ^15^ All studies were population-based or community-based (ie, did not recruit participants from clinics), although they had varying degrees of representativeness with regards to underlying populations of interest and some targeted a particular population (eg, the Religious Orders Study specifically sampled religious clergy; appendix pp 2–3). Reflecting the fact that deaths and most dementia in these countries occurs in those aged over 80 years, these cohorts had very small numbers of donors who died younger than 80 years, precluding the precise estimation of neuropathology prevalence in this age group. Therefore, we excluded these participants (ACT n=71 [9%], FHS n=42 [18%], CC75C n=1 [<1%], CFAS n=95 [17%], HAAS n=61 [8%], and ROSMAP n=180 [8%]); no other exclusions were made. All study protocols were approved by the ethics committees at each participating institution and all participants provided informed consent.

### Ascertainment of clinical dementia status

All studies designed procedures for dementia diagnosis in adherence with Diagnostic and Statistical Manual of Mental Disorders guidelines; however, implementation details varied. Diagnoses were made through consensus conference or were confirmed by a clinician in all studies except CFAS, which used an algorithmic approach.^16^ Those without clinical dementia at death and who did not have a study visit in the 2 years before death were assigned missing clinical dementia status in all studies except CFAS and CC75C, which included procedures for contacting family informants in such scenarios (appendix pp 4–5).

### Measurement of neuropathologies

Pathologists carrying out autopsies were masked to clinical dementia status. We assessed 12 commonly used neuropathology measures: arteriolosclerosis, atherosclerosis, macroinfarcts, microinfarcts, lacunes, cerebral amyloid angiopathy, Braak neurofibrillary tangle stage, Consortium to Establish a Registry for Alzheimer's disease (CERAD) diffuse plaque score, CERAD neuritic plaque score, hippocampal sclerosis, limbic-predominant age-related TDP-43 encephalopathy neuropathologic change (LATE-NC), and Lewy body pathology (appendix pp 10–15). For some measures, we used cut-points to dichotomise or coarsen data to maximise comparability between studies and ensure constructs were meaningful (appendix pp 7, 10–15). We considered Braak stage and CERAD plaque scores as markers of Alzheimer's disease, but acknowledge that Braak stage under V in the absence of amyloid **β** is considered diagnostic of primary age-related tauopathy, and the relationship between primary age-related tauopathy and Alzheimer's disease is an area of active research.^17^ Neuropathology measures were designed to be as consistent as possible over time within each study, although there were some modifications to the measurement of arteriolosclerosis, atherosclerosis and CERAD plaque scores in FHS.

### Statistical analysis

We examined characteristics of each contributing cohort using means and proportions. We compared the measurement of neuropathologies by examining differences in neuropathology protocols, and by quantifying differences across studies in the prevalence of neuropathologies by age group (80–89 years and ≥90 years). Initial analyses did not show large differences by sex; therefore, results were collapsed by sex to simplify the presentation of data. We excluded missing data (appendix p 6). On the basis of both the assessment of consistency in neuropathology prevalence and a qualitative assessment of standardisation in reported measurement procedures, we classified measures as having either low, moderate, or high confidence in harmonisation across studies. Because differences in neuropathology prevalence might be attributable to differences in sample characteristics, we gave more weight to the qualitative assessment of standardisation in final decisions.

To examine crude correlations between different neuropathologies, we calculated polychoric correlations between neuropathology measures within each study. The polychoric correlation estimates the correlation between two underlying latent constructs that have been assessed using a binary or ordinal measure.^18^ We assessed pair-wise unadjusted correlations within studies for each pair of available variables. Individuals with missing data were excluded (pair-wise deletion). We pooled correlations across studies using random-effects meta-analyses (appendix pp 16–29). We assessed statistical significance using an α level of 0·05.

To allow for the clear depiction of neuropathology co-occurrence, we limited co-occurrence analyses to six key neuropathology measures that were non-overlapping, captured different types of pathologies (Alzheimer's disease, vascular, or other), and had at least moderate confidence in the harmonisation of measures across cohorts: Braak stage, CERAD neuritic plaques, macroinfarcts, microinfarcts, LATE-NC stage, and Lewy bodies. Ordinal measures were dichotomised to create binary indicators of severe pathology (appendix p 7). We excluded records with missing data on any of the neuropathologies considered. Data from HAAS were not included because we did not have information available on CERAD neuritic plaques or LATE-NC. We used UpSet plots to visualise pathology co-occurrence stratified by clinical dementia status in the pooled data.^19^

The data required to re-weight findings to account for effects of selection into autopsy cohorts were not uniformly available for all studies, preventing comparable analyses across cohorts. Therefore, we selected the ACT cohort to investigate potential effects of selection on the prevalence of neuropathologies and the correlations between pathologies. We compared primary results in ACT with those accounting for selection using inverse probability of selection weighting (appendix pp 8–9).

All analyses were done using R (version 4.0.5).

### Role of the funding source

The funder of the study had no role in study design, data collection, data analysis, data interpretation, or writing of the report.

## Results

Cohorts included 4354 decedents aged 80 years or older with autopsy data, with between 190 (FHS) and 1988 (ROSMAP) decedents, and data collected between 1989 and 2022 (table). The average age at death was around 90 years in all samples (range 88·0 years [ACT] to 91·6 years [CC75C]), and greater than half of participants were women in all studies except HAAS, which only included men (table). Decedents in all cohorts were predominantly White with the exception of HAAS, which only included Japanese-American participants (table). The proportion of *APOE4* carriers ranged from 20% (142/700) in HAAS to 36% (81/227) in CC75C and the proportion of individuals with dementia ranged from 47% (827/1749) in ROSMAP to 66% (360/548) in HAAS (table).

**Table:** Sample characteristics of included cohorts (ROSMAP, ACT, CFAS, CC75C, FHS, and HAAS) and available neuropathologic measures in each cohort

		**ACT (n=755)**	**CC75C (n=241)**	**CFAS (n=467)**	**FHS (n=190)**	**HAAS (n=713)**	**ROSMAP (n=1988)**
Time period for autopsy collection		1996–2020	1998–2013	1989–2011	1996–2019	1992–2010	1994–2022
Age at death, years		88·0 (2·8)	91·6 (4·7)	89·6 (5·1)	90·6 (5·6)	88·7 (4·8)	90·6 (5·4)
Sex							
	Women	442 (59%)	170 (71%)	296 (63%)	112 (59%)	0	1375 (69%)
	Men	313 (41%)	71 (29%)	171 (37%)	78 (41%)	713 (100%)	613 (31%)
Race							
	White	712 (94%)	241 (100%)	467 (100%)	188 (99%)	0	1897 (96%)
	Black	9 (1%)	0	0	0	0	75 (4%)
	Other	34 (5%)	0	0	2 (1%)	713 (100%)	4 (<1%)
*APOE4* carriers		205/733 (28%)	81/227 (36%)	71/244 (29%)	45/174 (26%)	142/700 (20%)	470/1892 (25%)
Dementia		371/726 (51%)	127/230 (55%)	267/419 (64%)	90/188 (48%)	360/548 (66%)	827/1749 (47%)
Arteriolosclerosis		Yes	Yes	Yes	Yes	No	Yes
Atherosclerosis		Yes	Yes	Yes	Yes	No	Yes
Braak score		Yes	Yes	Yes	Yes	Yes	Yes
CERAD diffuse plaque score		No	Yes	Yes	Yes	No	Yes
CERAD neuritic plaque score		Yes	Yes	Yes	Yes	No	Yes
Cerebral amyloid angiopathy		Yes	Yes	Yes	Yes	Yes	Yes
Hippocampal sclerosis		Yes	Yes	Yes	Yes	Yes	Yes
Lacunes		No	Yes	Yes	Yes	No	Yes
LATE-NC		Yes	Yes	Yes	Yes	No	Yes
Lewy body disease		Yes	Yes	Yes	Yes	Yes	Yes
Macroinfarcts		Yes	Yes	Yes	Yes	Yes	Yes
Microinfarcts		Yes	Yes	Yes	Yes	Yes	Yes

Data are n (%), n/N (%), or mean (SD). Yes indicates measure available and no indicates measure not available. ACT=Adult Changes in Thought. CC75C=Cambridge City Over-75s Cohort Study. CERAD=Consortium to Establish a Registry for Alzheimer's disease. CFAS=Cognitive Function and Ageing Studies. FHS=Framingham Heart Study. HAAS=Honolulu-Asia Aging Study. LATE-NC=limbic-predominant age-related TDP-43 encephalopathy. ROSMAP=Religious Orders Study and Memory and Aging Project.

The consistency of measurements across cohorts varied widely (panel; appendix pp 10–15). For example, both ROSMAP and FHS assessed the severity of atherosclerosis in the circle of Willis (panel; appendix p 10). By contrast, ACT defined atherosclerosis as mild when restricted to the circle of Willis, but moderate when the disease was also found in other regions at the base of the brain, and severe when present in the cerebrum (panel; appendix p 10). CFAS and CC75C did not specify a location for the assessment of atherosclerosis (panel; appendix p 10).

Panel **Ascertainment methods for the measurement of atherosclerosis and Braak stage across included cohorts (ACT, CC75C, CFAS, FHS, HAAS, and ROSMAP)**

**Atherosclerosis**

*ACT*

Atherosclerosis was identified grossly by neuropathologists and was defined as mild when restricted to branch points in the circle of Willis, moderate when also in other regions at the base of the brain, and severe when present on the convexity of the cerebrum.

*CC75C*

Gross appearance and degree of atherosclerosis of large vessels. Graded as (none), 1 (mild), 2 (moderate), or 3 (severe).

*CFAS*

Gross appearance and degree of atherosclerosis of large vessels. Graded as 0 (none), 1 (mild), 2 (moderate), or 3 (severe).

*FHS*

Assessment changed in 2015; presence of atherosclerotic vascular pathology (of the circle or Willis, until 2014); severity of gross findings—atherosclerosis (of the circle of Willis, since 2015)

*HAAS*

Not assessed.

*ROSMAP*

Large vessel cerebral atherosclerosis rating by visual inspection at the circle of Willis at the base of the brain. Included assessment of the vertebral, basilar, posterior cerebral, middle cerebral, and anterior cerebral arteries and their proximal branches.

**Braak score**

*ACT*

By standardised methods (Braak, 1991).^20^

*CC75C*

By standardised methods (Braak, 1991).^20^

*CFAS*

By standardised methods (Braak, 1991).^20^ Where missing, neurofibrillary tangle scores were used to impute Braak stage category from limbic (hippocampus and entorhinal) and cortical (frontal, temporal, parietal, and occipital) areas. The pathology was graded as 0 (none), 1 (sparse; one or two affected neurons per section), 2 (moderate; several affected neurons per section), and 3 (severe; many affected neurons per section). Tangle density is referenced to images in the Consortium to Establish a Registry for Alzheimer's Disease Handbook.^21^

*FHS*

By standardised methods (Braak, 1991).^20^

*HAAS*

By standardised methods (Braak, 1991).^20^ Used Gallyas and Bielschowsky stained slides (× 20).

*ROSMAP*

By standardised methods (Braak, 1991).^20^

ACT=Adult Changes in Thought. CC75C=Cambridge City Over-75s Cohort Study. CFAS=Cognitive Function and Ageing Studies. FHS=Framingham Heart Study. HAAS=Honolulu-Asia Aging Study. ROSMAP=Religious Orders Study and Memory and Aging Project.

By comparison, although staining procedures can vary, ascertainment of Braak staging relied on mostly standardised methods in all studies. The protocol in CFAS deviated slightly; when complete Braak staging was missing, neurofibrillary tangle scores were used to impute Braak stage retrospectively, following previous publications (panel; appendix pp 10–11).^22^ In general, measures with the highest variation in ascertainment methods were those of vascular neuropathologies, whereas Alzheimer's disease measures were more standardised (panel; appendix pp 10–15).

**Figure 1:**
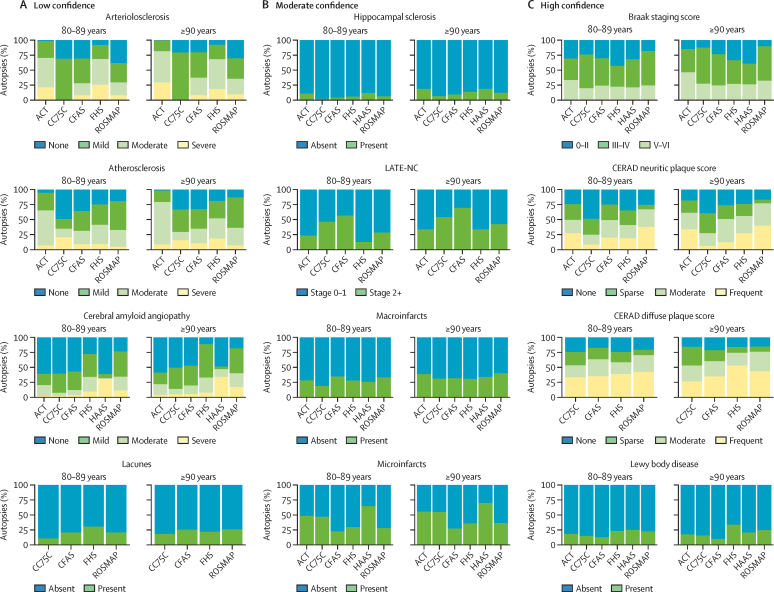
Crude prevalence and distributions of the 12 included neuropathologies across cohorts (ACT, CC75C, CFAS FHS, HAAS, and ROSMAP) by age group Cohorts were excluded from comparisons when they did not have data on a specific neuropathology. Neuropathologies are grouped into three groups (low, moderate, and high confidence) illustrating the confidence in harmonisation across cohorts based on an assessment of ascertainment methods and comparisons of crude prevalence. Arteriolosclerosis in CC75C is a binary measure and therefore the two categories correspond to absent and present. ACT=Adult Changes in Thought. CC75C=Cambridge City over-75s Cohort Study. CERAD=Consortium to Establish a Registry for Alzheimer's disease. CFAS=Cognitive Function and Ageing Studies. FHS=Framingham Heart Study. HAAS=Honolulu-Asia Aging Study. LATE-NC=limbic-predominant age-related TDP-43 encephalopathy neuropathologic change. ROSMAP=Religious Orders Study and Memory and Aging Project.

The level of consistency in measures across studies varied (figure 1). The most consistent measures across cohorts were Braak stage, CERAD diffuse plaque score, and Lewy body disease (figure 1). For example, the proportion of individuals aged 80–89 years with no Lewy body disease ranged from 13% to 25% (12 percentage points) across cohorts (figure 1). By comparison, the proportion of individuals aged 80–89 years with no cerebral amyloid angiopathy ranged from 24% to 61% (37 percentage points; figure 1). On the basis of comparisons of neuropathology prevalence and ascertainment methods, we grouped measures into those with low confidence (arterioloscerosis, atherosclerosis, cerebral amyloid angiopathy, and lacunes), moderate confidence (hippocampal sclerosis, LATE-NC, macroinfarcts, and microinfarcts), and high confidence (Braak stage, CERAD neuritic plaque score, CERAD diffuse plaque score, and Lewy body disease). Measures in the high confidence group largely were found in similar proportions across studies, although we found a lower burden of neuritic plaques in CC75C compared with in other cohorts (figure 1). Although details of administration might differ slightly (eg, specific methods of detection for Lewy bodies), all measures included in this category were based on highly standardised neuropathological criteria (Braak staging, CERAD guidelines, and modified McKeith criteria for Lewy bodies). Measures in the low and moderate categories showed less consistency across cohorts and we found noticeable differences in criteria, which could plausibly lead to differences in proportions (eg, atherosclerosis; figure 1).

Participants generally had a high burden of vascular and Alzheimer's disease neuropathologies. Across cohorts, a mean of 70% (SD 8) of individuals who died aged between 80–89 years had at least Braak stage III pathology; this proportion increased to 78% (SD 12) among those who died aged 90 years or older. Additionally, a mean of 40% (SD 16) of decedents aged 80–89 years and 47% (SD 16) of decedents aged 90 years or older in each study had some evidence of microinfarcts at death. By contrast, across cohorts, fewer decedents had evidence of hippocampal sclerosis (7% [SD 4] of those aged 80–89 years and 13% [SD 5] of those aged ≥90 years) or Lewy body pathology (19% [SD 5] of those aged 80–89 years and 20% [SD 8] of those aged ≥90 years).

**Figure 2:**
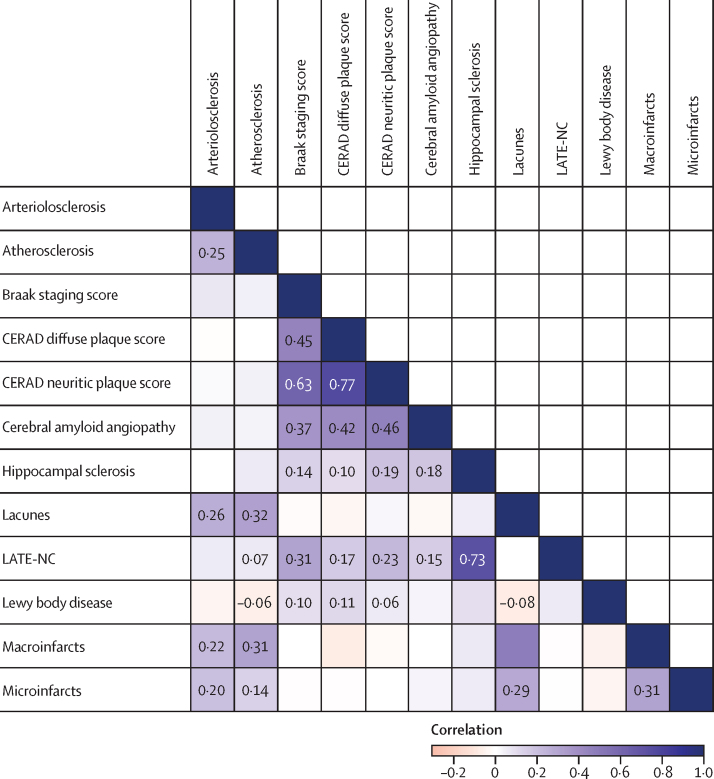
Correlation matrix describing inter-variability of neuropathological measures, summarised across cohorts (ACT, CC75C, CFAS FHS, HAAS, and ROSMAP) Correlations were first assessed within each cohort individually, and then pooled together using random-effects meta-analysis. Each cohort contributed to a given correlation when the pair of variables of interest in each correlation existed within a given dataset and were not perfectly collinear (no zero contingency cells). Numbers are shown when the correlation is statistically significant at the level of α=0·05. ACT=Adult Changes in Thought. CC75C=Cambridge City over-75s Cohort Study. CERAD=Consortium to Establish a Registry for Alzheimer's disease. CFAS=Cognitive Function and Ageing Studies. FHS=Framingham Heart Study. HAAS=Honolulu-Asia Aging Study. LATE-NC=limbic-predominant age-related TDP-43 encephalopathy neuropathologic change. ROSMAP=Religious Orders Study and Memory and Aging Project.

Alzheimer's disease neuropathology and vascular disease measures each had high correlations, creating two distinct clusters (figure 2; appendix p 4). Although occurring in the vasculature, cerebral amyloid angiopathy was more strongly correlated with Alzheimer's disease measures including Braak score (ρ=0·37 [95% CI 0·33–0·42) and CERAD neuritic plaque score (ρ=0·46 [0·34–0·57) than with vascular measures such as microinfarcts or macroinfarcts, which did not have significant correlations with cerebral amyloid angiopathy (figure 2; appendix p 4). Other than the correlation between CERAD diffuse and neuritic plaques, the strongest observed correlation was between LATE-NC and hippocampal sclerosis (ρ=0·73 [0·63–0·82]), which was expected because LATE-NC is thought be a common cause of hippocampal sclerosis (figure 2; appendix p 4). LATE-NC and Lewy bodies both had significant correlations with Alzheimer's disease neuropathologies, including Braak score (ρ=0·31 [0·20–0·42] for LATE-NC and ρ=0·10 [0·01–0·18] for Lewy bodies) and CERAD neuritic plaque score (ρ=0·23 [0·18–0·28] for LATE-NC and ρ=0·06 [0·01–0·11] for Lewy bodies; figure 2; appendix p 4).

**Figure 3:**
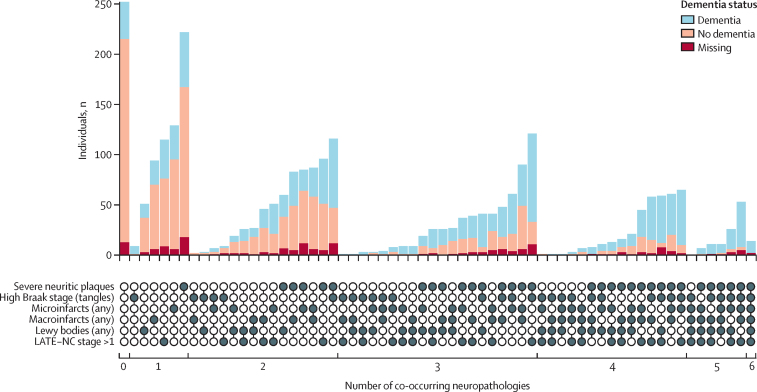
Co-occurrence of six key neuropathologies by clinical dementia status from data pooled across five cohorts (ACT, CC75C, CFAS FHS, and ROSMAP) Each bar represents the number of individuals with a unique combination of pathologies, as indicated by the filled in circles below. Individuals without dementia at death, who had a gap of greater than 2 years between the last study visit and death, were assigned a dementia status of missing in all studies except CFAS and CC75C, which included procedures to contact family informants in such circumstances. ACT=Adult Changes in Thought. CC75C=Cambridge City over-75s Cohort Study. CFAS=Cognitive Function and Ageing Studies. FHS=Framingham Heart Study. LATE-NC=limbic-predominant age-related TDP-43 encephalopathy neuropathologic change. ROSMAP=Religious Orders Study and Memory and Aging Project.

In data on six key neuropathologies (CERAD neuritic plaques, Braak stage, microinfarcts, macroinfarcts, Lewy bodies, and LATE-NC) pooled across five cohorts (ROSMAP, ACT, CFAS, CC75C, and FHS), 2443 (91%) of 2695 donors included in co-occurence analyses had at least one of these neuropathologies, 1823 (68%) had at least two, 1106 (41%) had at least three, 492 (18%) had at least four, 122 (5%) had five or six, and 14 (1%) had all six neuropathologies considered (figure 3; appendix pp 30–32). Clinical dementia status was related to the number of pathologies: 102 (93%) of 110 individuals with at least five neuropathologies had clinical dementia and 211 (26%) of 817 individuals with one or no neuropathologies had clinical dementia (figure 3; appendix pp 30–32). However, discordance was present; some individuals had a high number of co-occurring neuropathologies but no clinical dementia recorded before death and vice versa [(figure 3; appendix pp 30–32). Individuals with clinical dementia but without any included neuropathologies were those with dementia either due to another cause that was not considered in co-occurrence analyses, caused by neuropathological burden below the thresholds used for dichotomisation, or caused by neuropathologies not measured in this study. Of 764 decedents with severe cortical neuritic plaques and high Braak stages—the defining markers of Alzheimer's disease—only 116 (15%) did not have any other neuropathologies; 648 (85%) had at least one additional neuropathology, 377 (49%) had at least two, and 115 (15%) had three or four additional neuropathologies (figure 3; appendix pp 30–32). The most common additional pathology was LATE-NC, with 407 (53%) individuals with severe neuritic plaques and a high Braak stage also having LATE-NC stage greater than 1 (figure 3; appendix pp 30–32).

Results were similar in sensitivity analyses applying inverse probability of selection weighting approaches to the calculation of means and correlations to account for selection in the ACT autopsy sample (appendix pp 32–33).

## Discussion

Despite the challenges of harmonisation, we were able to assess the prevalence of neuropathologies by cohort, correlations between pathologies (assessed within each cohort and pooled across cohorts), and the co-occurrence of pathologies for six key measures of moderate or high confidence. Most decedents had Alzheimer's disease and vascular pathologies in their brains. The presence of different Alzheimer's disease pathologies or different vascular pathologies was highly correlated within these two clusters, but Alzheimer's disease and vascular measures were not correlated with each other. Significant proportions also had further neuropathological markers whether or not these individuals died with clinical dementia, despite a general association between burden of pathology and clinical dementia. In other words, these pathologies are common in the older population; within individuals they are mixed; and although related to dementia in life, many with these pathologies have died without dementia. This lack of determinism highlights gaps in our current understanding of the neuropathological and biological underpinnings of dementia in late life.

Findings on the crude prevalence of neuropathologies across cohorts are within the range of previous reports from individual or pooled population-based autopsy studies of LATE-NC, amyloid, tau, and vascular neuropathologies.^9^, ^23^, ^24^ The high observed burden of neuropathological features suggests that among those who die aged 80 years or older, the presence of some neuropathological burden is very common. The presence of Lewy body disease, hippocampal sclerosis, or macroinfarcts was less common compared with the high burden of small vessel vascular disease (arteriolosclerosis, cerebral amyloid angiopathy, and microinfarcts) and Alzheimer's disease neuropathologies. Our finding that neuropathologies commonly co-occurred replicates findings across many individual autopsy studies, which have shown that most individuals have more than one neuropathology simultaneously.^25^, ^26^

Our data showed that Alzheimer's disease neuropathology and vascular pathology were largely uncorrelated. Although a few published studies have found that atherosclerosis and arteriolosclerosis are associated with Alzheimer's disease neuropathologies,^27^, ^28^ results from most previous autopsy cohorts align with our findings.^29^, ^30^ In the context of existing evidence on vascular contributions to Alzheimer's disease,^31^, ^32^, ^33^, ^34^ findings suggest that vascular and Alzheimer's disease pathways might have independent causes, but interact synergistically to accelerate and promote cognitive decline and dementia. This interaction has potential consequences for the development of strategies to prevent and treat Alzheimer's disease and dementia, suggesting multifaceted approaches, targeting either an array of different lifestyle factors or multiple biological targets might be a more effective way to prevent disease and decline.^35^

Unlike vascular pathologies, LATE-NC did have significant correlations with Alzheimer's disease neuropathologies, including Braak stage and CERAD plaque scores. LATE-NC was also the most common additional pathology among individuals with high CERAD neuritic plaque scores and high Braak stages. Although smaller clinical studies have not consistently found significant associations between LATE-NC and Alzheimer's disease neuropathologies, our findings are in line with the largest existing study^9^ of LATE-NC, which pooled data across 13 autopsy cohorts. Potential mechanisms explaining the interrelatedness of TDP-43 (the protein that characterises LATE-NC) and Alzheimer's disease neuropathologies include reduced clearance of TDP-43 with the build-up of amyloid plaques or cross-seeding of amyloid-β with TDP-43.^36^ The connection between LATE-NC and Alzheimer's disease neuropathologies highlights the notion that Alzheimer's disease paradigms should incorporate more than amyloid and tau to accurately capture the underlying biological complexity. Proposals to extend the AT(N) framework (to classify Alzheimer's disease on the basis of biomarkers of amyloid-β [A], tau [T] and neurodegeneration [N]) to an ATX(N) system (where X represents biomarkers of other pathological mechanisms) integrating additional mechanisms move towards acknowledging and incorporating this complexity.^37^

Harmonised measures with high confidence were predominately measures of Alzheimer's disease neuropathologies, whereas vascular neuropathologies had lower confidence rankings. Our findings on the consistency of neuropathology measures and resultant confidence in harmonisation are a consequence of the availability and acceptance of standard norms by which to define and measure pathology. The assessment of neurofibrillary tangle distribution via Braak staging or the quantification of cortical neuritic and diffuse plaque densities using CERAD protocols (high confidence) were first introduced in the 1990s and are widely accepted. By contrast, similar standards do not exist for other neuropathologies, including vascular pathologies such as arteriolosclerosis (low confidence). The size requirement for lacunes (low confidence) varied, which affected definitional overlap and correlation with macroinfarcts; some studies even included lacunes in their definitions of macroinfarcts (ACT, FHS, and ROSMAP). Our process for determining harmonisation confidence necessarily relied on qualitative assessments of measure standardisation; therefore, findings are not based completely on objective criteria. However, our conclusions align with previous findings from a survey of neuropathologists from 22 members of the BrainNet Europe consortium, which also highlighted existing variability in the assessment and the quantification of vascular neuropathologies.^38^ To allow for high-quality harmonisation efforts and comparisons between studies, additional efforts are needed to develop frameworks for the measurement of vascular neuropathologies such as arteriolosclerosis, atherosclerosis, and lacunes.

Published consensus criteria for LATE-NC (moderate confidence) helped facilitate consistent measurement; although, the post-hoc application of these criteria to existing cohorts with different protocols for the measurement of TDP-43 (including differences in sampling and TDP-43 antibodies) might have hindered comparability.^39^ As criteria continue to evolve, to ensure the consistency of implementation across centres in future studies, details on recommended sampling and staining procedures should be included in guidelines when such details are likely to affect measurement.

Strengths of this study include the combination and comparison of six large community-based autopsy studies, and the inclusion of various neuropathologies, including the highly prevalent LATE-NC, which was only defined^39^ in 2019 and has not been included in most previous studies. Our study has some limitations. First, one must consider how selection processes surrounding consent to autopsy might affect sample composition. In a sensitivity analysis in ACT, we used inverse probability weighting to correct potential bias due to selection and findings for neuropathology prevalence and correlations were unchanged; however, we did not examine effects on associations with dementia. Similar efforts to adjust for selection bias in FHS and CFAS have also yielded similar results to those of the primary analyses and analyses in HAAS showed that those who consented and those who did not consent to autopsy were similar with regards to most characteristics.^14^, ^40^, ^41^ Second, we excluded individuals with missing data from analyses. However, for both the assessment of neuropathology prevalence and correlation, we included all individuals with non-missing data on the specific pathologies of interest to limit the number of excluded records. Additionally, levels of missing data were generally low, and higher levels of missing data (LATE-NC in FHS) could often be explained by the post-hoc addition of neuropathologies, leading to earlier autopsy cases not being assessed. In this case, missingness would probably be uninformative unless strong cohort effects are present. Third, between-study variation reduces the comparability between the included studies. To help address this concern, we created our categorisations of harmonisation confidence to help contextualise findings and highlight important issues in the measurement of neuropathologies. Despite similarity in the general criteria, differences in procedures for dementia ascertainment might also have some effect.

Fourth, limitations of using autopsy data should be considered. Individuals who come to autopsy are only observed at the end of their lives, and their pathologies are likely to be different from those still alive. Although in vivo biomarkers such as tau-PET and amyloid-PET or novel CSF and blood biomarkers circumvent these issues and allow for the assessment of neuropathologies at younger ages, such biomarkers bring a new set of challenges including measurement error, problems with selection bias, and feasibility in old-age populations. Therefore, thus far such measures have not been administered in large, community-based studies. Although the included studies varied in terms of their representativeness to underlying populations of interest (ROSMAP specifically recruited religious clergy), none of the included studies sampled individuals from clinical settings. Fifth, we were unable to include all possible neuropathologies and descriptions of neuropathology co-occurrence were limited to six key neuropathologies. However, other neuropathologies exist and individuals with zero neuropathologies in the co-occurrence analyses we present here might have neuropathologies that were not included. Although we included various neuropathologies in the broader analysis, this study was not exhaustive with respect to the potential neuropathologies included; future work should add new neuropathologies or include more specific categorisations (eg, cortical *vs* subcortical Lewy bodies). Finally, the studies included were from high-income countries and, with the exception of HAAS, participants were predominantly White and well educated.^10^, ^11^, ^12^, ^13^, ^14^, ^15^ Given evidence of differences in the prevalence of neuropathologies by factors such as race or socioeconomic status,^42^ additional work in more diverse settings and samples is warranted.

This analysis provides compelling evidence that should inform the way we think about dementia in ageing populations. Policies and investments are typically framed around single subtypes of dementia. Practice and research are commonly predicated on the assumption of a deterministic relationship between pathology and the clinical expression of dementia. In line with previous individual studies, findings from this combined analysis question this paradigm. Given the combination of pathologies in the brains of people with dementia at old ages, single therapies might be unlikely to have large effects.

Despite clear benefits of pooling data across autopsy studies, including increased power and the ability to make comparisons across settings, substantial barriers to harmonisation exist. The development of accepted standards for comparable measurement across neuropathologies, in particular for vascular neuropathologies, would constitute an important step forward and allow for more nuanced analyses. Despite harmonisation challenges, our data highlighted high levels of neuropathologic burden, separate clustering of Alzheimer's disease and vascular neuropathologies, the high degree of neuropathology co-occurrence, and the association between multiple pathologies and clinical dementia. Questions remain regarding the lack of determinism between pathology and clinical outcomes. Researchers must find new and innovative ways to leverage existing valuable data (eg, data pooling and harmonisation) and the research field must pursue goals that acknowledge the complexity and multi-morbidity of brain pathology in the general old-age population.

## Data sharing

Data from FHS, HAAS, CC75C, and CFAS are not publicly available or available via application because of data protection and security reasons. Data from the ROSMAP study are available from https://www.radc.rush.edu/ following a project-proposal approval process. Data from the ACT study are similarly available following a proposal approval at https://actagingresearch.org/resources/act-cohort.

## Declaration of interests

SS reports consulting fees from Biogen and Eisai. JS reports consulting fees from AVID, Alnylam Pharmaceuticals, and Cerveau Technologies. All other authors declare no competing interests.

## References

[1] Nichols E, Steinmetz JD, Vollset SE (2022). Estimation of the global prevalence of dementia in 2019 and forecasted prevalence in 2050: an analysis for the Global Burden of Disease Study 2019. Lancet Public Health.

[2] Ferrari C, Sorbi S (2021). The complexity of Alzheimer's disease: an evolving puzzle. Physiol Rev.

[3] Cummings J, Reiber C, Kumar P (2018). The price of progress: Funding and financing Alzheimer's disease drug development. Alzheimers Dement (N Y).

[4] Ackley SF, Zimmerman SC, Brenowitz WD (2021). Effect of reductions in amyloid levels on cognitive change in randomized trials: instrumental variable meta-analysis. BMJ.

[5] Mullane K, Williams M (2020). Alzheimer's disease beyond amyloid: Can the repetitive failures of amyloid-targeted therapeutics inform future approaches to dementia drug discovery?. Biochem Pharmacol.

[6] Crane PK, Gibbons LE, McCurry SM (2016). Importance of home study visit capacity in dementia studies. Alzheimers Dement.

[7] Crane PK, Gibbons LE, Dams-O'Connor K (2016). Association of traumatic brain injury with late-life neurodegenerative conditions and neuropathologic findings. JAMA Neurol.

[8] Keage HAD, Ince PG, Matthews FE, Wharton SB, McKeith IG, Brayne C (2012). Impact of less common and “disregarded” neurodegenerative pathologies on dementia burden in a population-based cohort. J Alzheimers Dis.

[9] Nelson PT, Brayne C, Flanagan ME (2022). Frequency of LATE neuropathologic change across the spectrum of Alzheimer's disease neuropathology: combined data from 13 community-based or population-based autopsy cohorts. Acta Neuropathol.

[10] Sonnen JA, Larson EB, Haneuse S (2009). Neuropathology in the adult changes in thought study: a review. J Alzheimers Dis.

[11] Tsao CW, Vasan RS (2015). Cohort Profile: The Framingham Heart Study (FHS): overview of milestones in cardiovascular epidemiology. Int J Epidemiol.

[12] Fleming J, Zhao E, O'Connor DW, Pollitt PA, Brayne C (2007). Cohort profile: the Cambridge City over-75s Cohort (CC75C). Int J Epidemiol.

[13] Brayne C, McCracken C, Matthews FE (2006). Cohort profile: the Medical Research Council Cognitive Function and Ageing Study (CFAS). Int J Epidemiol.

[14] Gelber RP, Launer LJ, White LR (2012). The Honolulu-Asia Aging Study: epidemiologic and neuropathologic research on cognitive impairment. Curr Alzheimer Res.

[15] Bennett DA, Buchman AS, Boyle PA, Barnes LL, Wilson RS, Schneider JA (2018). Religious Orders Study and Rush Memory and Aging Project. J Alzheimers Dis.

[16] Dewey ME, Copeland JRM (1986). Computerized psychiatric diagnosis in the elderly: AGECAT. J Microcomput Appl.

[17] Besser LM, Mock C, Teylan MA, Hassenstab J, Kukull WA, Crary JF (2019). Differences in cognitive impairment in primary age-related tauopathy versus Alzheimer disease. J Neuropathol Exp Neurol.

[18] Olsson U (1979). Maximum likelihood estimation of the polychoric correlation coefficient. Psychometrika.

[19] Lex A, Gehlenborg N, Strobelt H, Vuillemot R, Pfister H (2014). UpSet: Visualization of intersecting sets. IEEE Trans Vis Comput Graph.

[20] Braak H, Braak E (1991). Neuropathological stageing of Alzheimer-related changes. Acta Neuropathol.

[21] Mirra SS, Heyman A, McKeel D (1991). The Consortium to Establish a Registry for Alzheimer's Disease (CERAD). Part II. Standardization of the neuropathologic assessment of Alzheimer's disease. Neurology.

[22] Hokkanen SRK, Hunter S, Polvikoski TM (2018). Hippocampal sclerosis, hippocampal neuron loss patterns and TDP-43 in the aged population. Brain Pathol.

[23] Wharton SB, Brayne C, Savva GM (2011). Epidemiological neuropathology: the MRC Cognitive Function and Aging Study experience. J Alzheimers Dis.

[24] Kovacs GG, Alafuzoff I, Al-Sarraj S (2008). Mixed brain pathologies in dementia: the BrainNet Europe consortium experience. Dement Geriatr Cogn Disord.

[25] Bennett DA, Wilson RS, Arvanitakis Z, Boyle PA, de Toledo-Morrell L, Schneider JA (2013). Selected findings from the Religious Orders Study and Rush Memory and Aging Project. J Alzheimers Dis.

[26] Brayne C, Richardson K, Matthews FE (2009). Neuropathological correlates of dementia in over-80-year-old brain donors from the population-based Cambridge city over-75s cohort (CC75C) study. J Alzheimers Dis.

[27] Honig LS, Kukull W, Mayeux R (2005). Atherosclerosis and AD: analysis of data from the US National Alzheimer's Coordinating Center. Neurology.

[28] Kapasi A, Yu L, Petyuk V, Arfanakis K, Bennett DA, Schneider JA (2022). Association of small vessel disease with tau pathology. Acta Neuropathol.

[29] Dolan H, Crain B, Troncoso J, Resnick SM, Zonderman AB, OBrien RJ (2010). Atherosclerosis, dementia, and Alzheimer's disease in the BLSA cohort. Ann Neurol.

[30] Launer LJ, Petrovitch H, Ross GW, Markesbery W, White LR (2008). AD brain pathology: vascular origins? Results from the HAAS autopsy study. Neurobiol Aging.

[31] Nagy Z, Esiri MM, Jobst KA (1997). The effects of additional pathology on the cognitive deficit in Alzheimer disease. J Neuropathol Exp Neurol.

[32] Snowdon DA, Greiner LH, Mortimer JA, Riley KP, Greiner PA, Markesbery WR (1997). Brain infarction and the clinical expression of Alzheimer disease. The Nun study. JAMA.

[33] White L, Petrovitch H, Hardman J (2002). Cerebrovascular pathology and dementia in autopsied Honolulu-Asia Aging Study participants. Ann N Y Acad Sci.

[34] Kapasi A, Leurgans SE, Arvanitakis Z, Barnes LL, Bennett DA, Schneider JA (2021). Aβ (amyloid beta) and tau tangle pathology modifies the association between small vessel disease and cortical microinfarcts. Stroke.

[35] Kivipelto M, Mangialasche F, Snyder HM (2020). World-Wide FINGERS Network: a global approach to risk reduction and prevention of dementia. Alzheimers Dement.

[36] Xu G, Stevens SM, Moore BD, McClung S, Borchelt DR, Borchelt DR (2013). Cytosolic proteins lose solubility as amyloid deposits in a transgenic mouse model of Alzheimer-type amyloidosis. Hum Mol Genet.

[37] Hampel H, Cummings J, Blennow K, Gao P, Jack CR, Vergallo A (2021). Developing the ATX(N) classification for use across the Alzheimer disease continuum. Nat Rev Neurol.

[38] Alafuzoff I, Gelpi E, Al-Sarraj S (2012). The need to unify neuropathological assessments of vascular alterations in the ageing brain: multicentre survey by the BrainNet Europe consortium. Exp Gerontol.

[39] Nelson PT, Dickson DW, Trojanowski JQ (2019). Limbic-predominant age-related TDP-43 encephalopathy (LATE): consensus working group report. Brain.

[40] Conner SC, Pase MP, Carneiro H (2019). Mid-life and late-life vascular risk factor burden and neuropathology in old age. Ann Clin Transl Neurol.

[41] Matthews FE, Brayne C, Lowe J, McKeith I, Wharton SB, Ince P (2009). Epidemiological pathology of dementia: attributable-risks at death in the Medical Research Council Cognitive Function and Ageing Study. PLoS Med.

[42] Barnes LL, Leurgans S, Aggarwal NT (2015). Mixed pathology is more likely in black than white decedents with Alzheimer dementia. Neurology.

