# Supplementary material for: The prevalence, correlation, and co-occurrence of neuropathology in old age: harmonisation of 12 measures across six community-based autopsy studies of dementia
Source: Lancet Healthy Longev. 2023 Mar;4(3):e115–25. doi: 10.1016/S2666-7568(23)00019-3 (PMC9977689; doi:10.1016/S2666-7568(23)00019-3)
Supplement: Supplementary appendix [file mmc1.pdf]

THE LANCET
Healthy Longevity

Supplementary appendix

This appendix formed part of the original submission and has been peer reviewed. We post it as supplied by the authors.

Supplement to: Nichols E, Merrick R, Hay SI, et al. The prevalence, correlation, and co-occurrence of neuropathology in old age: harmonisation of 12 measures across six community-based autopsy studies of dementia. *Lancet Healthy Longev* 2023;
4: e115–25.

Appendix

Table of Contents

Appendix 1: Cohort descriptions 2

Appendix 2: Dementia Ascertainment by Cohort 4

Figure S1. Missing data on neuropathologies for each cohort 6

Appendix 3: Dichotomization of six key neuropathologies included in UpSet plots 7

Appendix 4: Methodological details on analyses to account for selection bias in the ACT cohort 8

Appendix Table S1. Ascertainment methods across cohorts for all neuropathologies considered 10

Appendix 5: Meta-analyses of polychoric correlations to summarize across cohorts 16

Appendix Table S2. Numbers of individuals with and without dementia for different combinations of neuropathology co-occurrence 30

Appendix Figure S4. Comparison of weighted and unweighted estimates of the distribution of neuropathologic burden in the ACT study 32

Appendix Figure S5. Comparison of weighted and unweighted estimates of the correlations between neuropathologies in the ACT study 33

Appendix 5: STROBE Checklist 34

Appendix 1: Cohort descriptions

Adult Changes in Thought (ACT) Study: ACT was started in 1994, and recruits participants who are members of the Kaiser Permanente Washington health plan (previously Group Health), who are over 65, do not have dementia, do not reside in a nursing home, and who have been enrolled in the health plan for at least two years. Participants are roughly reflective of the population of King County, Washington. In addition to the initial cohort, an expansion cohort, and an ongoing replacement cohort were added. Ongoing recruitment targets a stable cohort of ~2,000 participants. About a third of participants who have died have consented to brain autopsy.

Framingham Heart Study (FHS): Participants of the Framingham Heart Study (FHS) were originally recruited in 1948 based via mailed invitation letters based on random sampling of 2/3 of families with members aged 30-59 living in Framingham MA, USA. Additional cohorts, including the Offspring and Third Generation cohorts have created and added to the intergenerational nature of the study. The autopsy program began in 1997 and is voluntary. Autopsy data are available for selected participants in the Original and Offspring cohorts.

Cambridge City over-75s Cohort Study (CC75C): The Cambridge City over-75s Cohort Study targeted all individuals 75 years and old who were registered at selected general medical practices in Cambridge, England, UK. General practices were selected to be geographically and socially representative of the city. The original survey in 1985 had a response rate of 95%, and individuals have been followed for 28 years. Although recruitment for the brain autopsy program initially focused on those with lower cognition, subsequent efforts were made to add individuals with normal cognitive functioning and the distribution of Mini-Mental State Examination (MMSE) scores in the autopsy sample closely matches the distribution in the full cohort at baseline.

Cognitive Functioning and Ageing Studies (CFAS): The CFAS study had five centers in England and Wales (Cambridgeshire, Gwynedd, Newcastle upon Tyne, Nottingham, and Oxford). Random samples of 2500 participants aged 64 years and older were sampled. An additional 5200 individuals were included from a sixth center based in Liverpool. The study included two waves (four in Liverpool), and participants were invited to participant in a brain donation program.

Honolulu-Asia Aging Study (HAAS): The Honolulu-Asia Aging Study (HAAS; baseline 1990) includes 80% surviving members of the existing Honolulu Heart Program (HHP), which started in 1965 as a community-based cohort study of cardiovascular disease. The original cohort included all Japanese-American men born between 1900 and 1919 and listed on the Honolulu Selective Service rolls for World War II and still living on Oahu, Hawaii at baseline. The autopsy study was started in 1992, when an invitation to participate was offered to all men regardless of dementia diagnosis. Analysis of brain pathology followed a standardized protocol.

The Religious Orders Study and Memory and Aging Project (ROSMAP): The Religious Orders Study (ROS) began in 1994 and enrolls nuns, priests, and brothers from across the US. The Memory and Aging Project started in 1997 and includes lay persons from across northeastern Illinois recruited from retirement communities. Both studies are ongoing with continuing recruitment and require consent to autopsy at study entry. Both studies also have biannual data collection and protocols and data collection

methods are standardized. The two studies are often analyzed together and are collectively known as ROSMAP.

Appendix 2: Dementia Ascertainment by Cohort

Adult Changes in Thought (ACT) Study: All participants were assessed with the Cognitive Abilities Screening Instrument (CASI) at biennial study visits. Individuals who scored lower than 86 on the CASI or those who were referred due to staff or family concerns underwent comprehensive neuropsychological testing and detailed physical and neurological examinations. Dementia diagnoses were made after thorough medical records review and discussion of examinations via consensus conference. Diagnoses were based on the Diagnostic and Statistical Manual of Mental Disorders (DSM)-IV criteria.

Framingham Heart Study (FHS): At every biannual study visit, participants are administered the Mini-Mental State Examination (MMSE). Participants are flagged for further testing in a number of different ways: a score < 23 for anyone, <24 for persons with a high school education, < 26 for college-educated participants, decline of 3 points from a prior exam, decline of 5 points from person best score. Participants may also be flagged by themselves, their family or their physician. If flagged, participants receive a full neuropsychological battery and a neuropsychologist assigns a severity score based on this testing. Participants flagged in this stage then receive a full neurologic exam, and those who are flagged in this stage are considered by a dementia review panel. The review panel (consisting of a neurologist and neuropsychologist) makes a definition of dementia based on DSM-IV criteria.

Cambridge City over-75s Cohort Study (CC75C): All study participants initially completed the MMSE. Those with low to moderate scores (0-23) and ¼ of participants with mild or minimal cognitive impairment (24-25) received more in-depth interviews via the Cambridge Diagnostic Examination for the Elderly (CAMDEX), which was administered by a psychiatrist and includes a proxy informant interview. At death, two clinicians agreed on a consensus diagnosis consistent with DSM-IV criteria following review of all interviews, proxy informant reports, death certificates and retrospective interviews with relatives following the participant's death.

Cognitive Functioning and Ageing Studies (CFAS): The screening interview included basic questions on demographics, health, functional impairment, and cognition (the MMSE). All individuals who were either suspected of having dementia or who had an MMSE less than or equal to 21, ½ to 1/10 of individuals who were not suspected of dementia and had MMSE scores above 21 were invited for a more comprehensive assessment using the Geriatric Mental State (GMS) examination. The information in the GMS examination was used in the Automated Geriatric Examination for Computer Assisted Taxonomy (AGECAT) algorithm. The GMS-AGECAT algorithm has been validated internationally against DSM-III-R criteria. When missing data precluded the use of the GMS-AGECAT algorithm, diagnosis was given by a clinician after review of all available information.

Honolulu-Asia Aging Study (HAAS): At every examination, the 100-point Cognitive Abilities Screening Instrument (CASI) was used to assess cognitive functioning. At baseline, individuals with poor CASI score (<74) as well as stratified samples of participants with higher scores received a full dementia evaluation, including proxy interview. At later visits, only those with low CASI scores received a full dementia evaluation. When it was not possible to complete a full evaluation, CASI score of <65 was used to signal definite cognitive impairment, 65-73 marginal cognitive impairment, 74-82 low-normal and greater than 82 normal. The more comprehensive dementia evaluations included a neurological examination, -

neuropsychological testing, and an informant interview. A consensus diagnosis of dementia was based on DSM III-R criteria.

The Religious Orders Study and Memory and Aging Project (ROSMAP): At each annual examination, all participants underwent a comprehensive clinical assessment and neuropsychological battery. Education-stratified cutoffs for 11 cognitive tests covering 5 cognitive domains were developed and used to assess cognitive impairment. These impairment ratings along with other cognitive tests scores, basic demographics, and information on sensory and motor deficits or difficulties during cognitive testing were reviewed by neuropsychologists to come to a decision regarding the presence of clinical impairment in each of the 5 cognitive domains. A clinician then reviewed decisions on impairment status along with all other relevant clinical information to come to a clinical judgement on the presence of dementia. At the time of death, all relevant clinical information over time (but no information based on autopsy assessments) was re-reviewed to come to a final decision on clinical status at death.

Figure S1. Missing data on neuropathologies for each cohort

Missing data is relatively low for most measures across the majority of cohorts, with some exceptions. Microinfarcts, macroinfarcts, and cerebral amyloid angiopathy were available in a subset of individuals in HAAS. LATE-NC stage was only assessed in a subset of individuals in FHS.

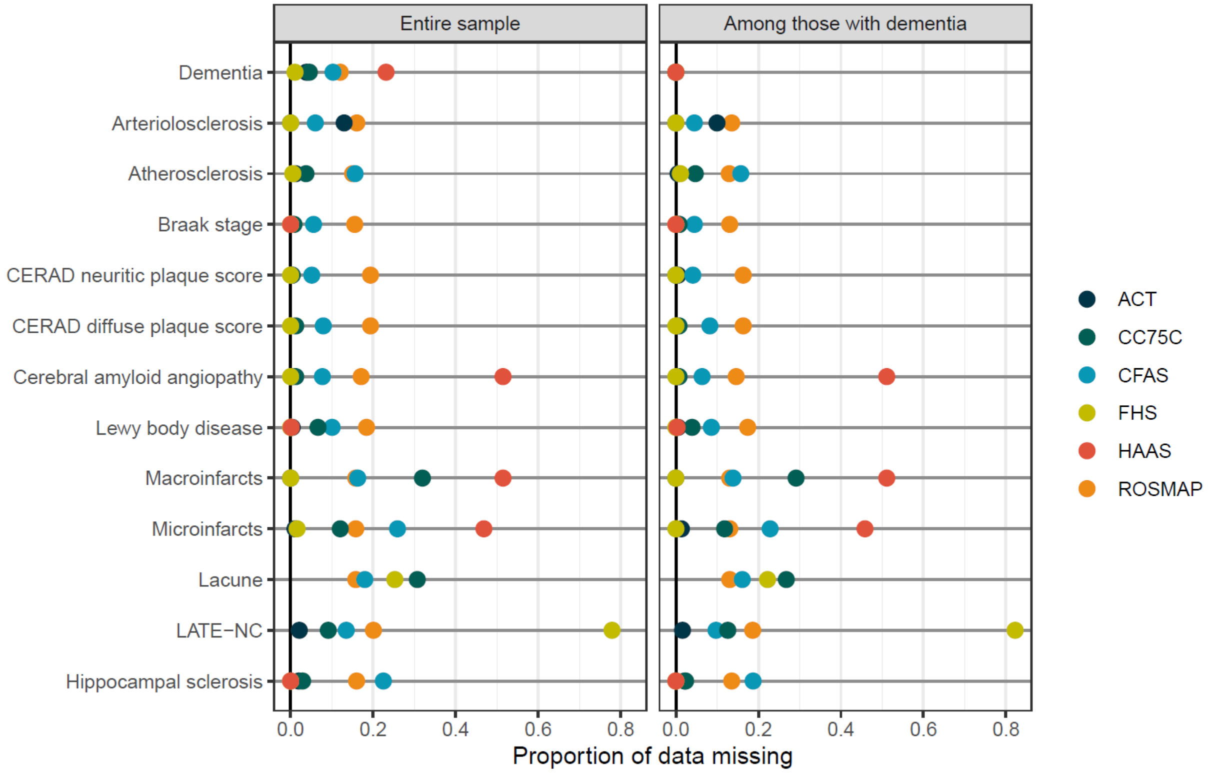

Appendix 3: Dichotomization of six key neuropathologies included in UpSet plots

Braak Stage: Present corresponds to Braak stage of V or VI

CERAD neuritic plaque score: Present corresponds to CERAD score of moderate or severe

Macroinfarcts: Present corresponds to any macroinfarcts present

Microinfarcts: Present corresponds to any microinfarcts present

LATE-NC stage: Present corresponds to LATE-NC stage of 2 or greater

Lewy bodies: Present corresponds to any Lewy body pathology present

Appendix 4: Methodological details on analyses to account for selection bias in the ACT cohort

Those who choose to consent to autopsy may be different from those who do not consent. While we had access to data on those who consented, the inferences and conclusions we want to make are about the entire cohort. In all cohorts except ROSMAP (which required consent to autopsy for study entry), only a subset of participants agreed to autopsy. To test the potential effects of this bias, we used inverse probability weighting to account for this potential source of bias in the ACT cohort, which contained the information necessary to construct inverse probability weights.

In ACT, we first identified variables that could plausibly be associated with consent to autopsy, and which could influence our findings and conclusions. The variables we considered were:

- Age (& Age^2 to capture nonlinear associations)
- Gender
- Education (yrs)
- Dementia
- Self-reported Hispanic ethnicity
- Nursing home residence (yes/no)
- Marital status (never married/married/separated or divorced/other)
- Smoking status (never smoker/past smoker/current smoker)
- Stroke (self-reported)
- Heart disease (myocardial infarction, angina, CABG, angioplasty) (self-reported)
- Diabetes (self-reported)
- Center for epidemiologic studies - depression scale (CESD) (per point)

We estimated a logistic regression to predict consent to autopsy among individuals who had died (and therefore were eligible to be able to contribute autopsy data) using the above variables. We used the missing indicator method to account for missing data in the predictors of consent to autopsy.[1] Although this method can lead to bias when trying to estimate the effect of an exposure on an outcome, because our primary goal is prediction, we expect meaningful error to be minimal. We estimated weights in those who consented to autopsy as:

$$Weight = \frac{Proportion\ consenting\ to\ autopsy}{Predicted\ probability\ of\ consenting\ to\ autopsy}.$$

The mean of the weights was 1.00, with a range from 0.36-8.23. We evaluated the performance of the weights by contrast the balance between the full sample and the autopsy sample using standardized mean differences for continuous variables and differences in proportions for binary variables. We assessed differences both before and after weighting. Weighting achieved balance on all of the predictors considered.

Figure S2. Assessment of balance on key predictors before and after inverse probability weighting to account for selection into the autopsy cohort in the ACT sample

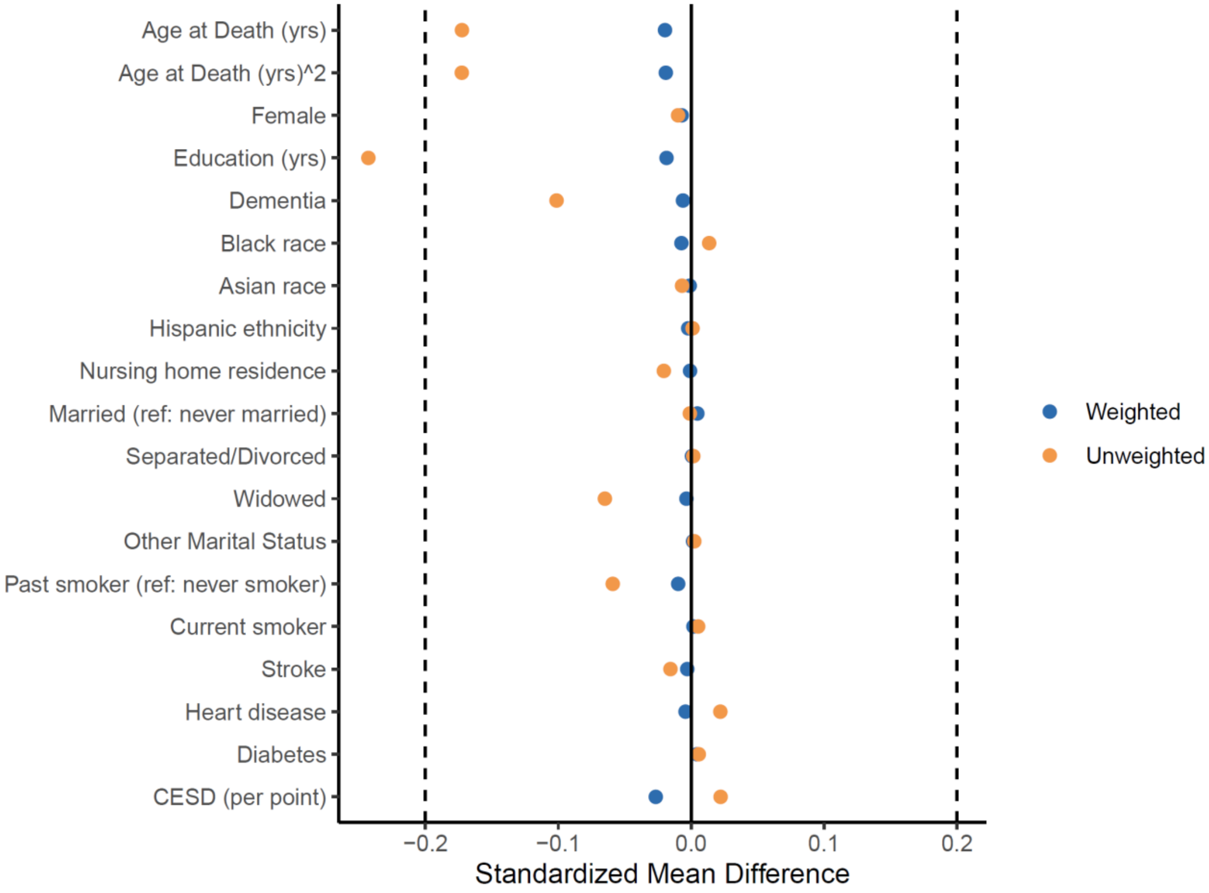

References

1. Greenland S, Finkle WD. A Critical Look at Methods for Handling Missing Covariates in Epidemiologic Regression Analyses. American Journal of Epidemiology 1995; 142: 1255–64.

Appendix Table S1. Ascertainment methods across cohorts for all neuropathologies considered

Measure	Study	Definition
Arteriolosclerosis	RUSH	Visual assessment and rating of histological changes (intimal deterioration, smooth muscle degeneration, and fibrohyalinotic thickening of arterioles with consequent narrowing of vascular lumen).
	ACT	A rating of none indicates that the thickness is appropriate for the vessel size and there isn't significant damage to the surrounding brain parenchyma. Mild indicates a slight thickening of the vessel wall with notable damage to surrounding parenchyma. Moderate indicates obvious thickening with some loss of endothelial cells and slight narrowing of the lumen. Severe corresponds to "onion-skinning" of the vessels, meaning there is layering of collagen in the vessel wall, leading to marked narrowing of the lumen.
	CFAS	Severity of microvascular arteriolosclerosis. Graded as none = 0, mild = 1, moderate = 2, severe = 3.
	CC75C	(Binary) Presence or absence of severe microvascular arteriolosclerosis
	Framingham	Assessment addressed the following questions: "Is arteriosclerosis (small parenchymal arteriolar disease) present? (Data available through 2014); Arteriolosclerosis? (Assessed in subcortical white or gray matter) (data available since 2015)
	HAAS	Not assessed.
Atherosclerosis	RUSH	Large vessel cerebral atherosclerosis rating by visual inspection at the Circle of Willis at the base of the brain. Included evaluation of the vertebral, basilar, posterior cerebral, middle cerebral, and anterior cerebral arteries and their proximal branches.
	ACT	Atherosclerosis was identified grossly by neuropathologists. This was defined as "mild" when restricted to branch points in the circle of Willis, "moderate" when also in other regions at the base of the brain, and "severe" when present on the convexity of the cerebrum)
	CFAS	Gross appearance and degree of atherosclerosis of large vessels. Graded as none = 0, mild = 1, moderate = 2, severe = 3.
	CC75C	Gross appearance and degree of atherosclerosis of large vessels. Graded as none = 0, mild = 1, moderate = 2, severe = 3.
	Framingham	Assessment addressed the following questions: Is atherosclerotic vascular pathology (of the circle or Willis) present? (through 2014); Severity of gross findings - Atherosclerosis (of the circle of Willis)? (since 2015)
	HAAS	Not assessed.
Braak Score	RUSH	By standardized methods (Braak & Braak, 1991)
	ACT	By standardized methods (Braak & Braak, 1991)

	CFAS	By standardized methods (Braak & Braak, 1991). Where missing, NFT scores used to impute Braak stage category (described in Notes) from limbic (hippocampus, entorhinal) and cortical (frontal, temporal, parietal, occipital) areas. The pathology is graded as none = 0, sparse (one or two affected neurons per section) = 1, moderate (several affected neurons per section) = 2 and severe (many affected neurons per section) = 3. Tangle density is referenced to images in CERAD Handbook.
	CC75C	By standardized methods (Braak & Braak, 1991)
	Framingham	By standardized methods (Braak & Braak, 1991)
	HAAS	By standardized methods (Braak & Braak, 1991). Used Gallyas and Bielschowsky stained slides (20x).
CERAD Neuritic Plaques	RUSH	Semi-quantitative scores (0-5 range) were generated in the frontal cortex, superior temporal cortex, entorhinal cortex, hippocampus CA1, and inferior parietal cortex. Scores of 0 were labeled none, a score of 1-3 was labeled mild, a score of 4 was labeled moderate, and a score of 5 was labeled severe.
	ACT	By standardized methods (Mirra et al., 1991)
	CFAS	By standardized methods (Mirra et al., 1991). Maximum cortical neuritic plaque score based on severity of tau reactive neuritic plaques in the frontal, temporal, parietal and occipital cortices. The pathology is graded as none = 0, sparse (one or two plaques per section) = 1, moderate (several plaques per section) = 2 and severe (many plaques per section) = 3. Plaque density is referenced to images in CERAD Handbook.
	CC75C	By standardized methods (Mirra et al., 1991). Maximum cortical neuritic plaque score based on severity of tau reactive neuritic plaques in the frontal, temporal, parietal and occipital cortices. The pathology is graded as none = 0, sparse (one or two plaques per section) = 1, moderate (several plaques per section) = 2 and severe (many plaques per section) = 3. Plaque density is referenced to images in CERAD Handbook.
	Framingham	CERAD Neuritic plaques (plaques with argyrophilic dystrophic neuritis with or without dense amyloid cores) (through 2014). CERAD score for density of neocortical neuritic plaque (plaques with argyrophilic dystrophic neurites, with or without dense amyloid cores) (since 2015)
	HAAS	Variable not available.
Cerebral Amyloid Angiopathy	RUSH	Semi-quantitative scores were generated for four regions (midfrontal, midtemporal, parietal, and calcarine cortices) in both meningeal and parenchymal vessels. The maximum of the meningeal and parenchymal scores were taken and averaged to create a continuous measure of CAA pathology. Then cutoffs were determined by neuropathologists to create an overall ordinal scale.
	ACT	Rated as none, mild, moderate, severe according to staging by Greenberg & Vonsattel (1997). Only assessed the occipital lobe.
	CFAS	Overall average cortical score for CAA (based on maximum scores for either parenchymal or meningeal CAA for each of the four cortical areas) converted back to categorical measure (similar to Love et al. 2014). Severity of vascular amyloid deposits in the brain parenchyma/meninges of frontal, temporal, parietal, and occipital cortices respectively. The pathology is graded as none = 0, sparse (one or two affected vessels per section) =1, moderate (several vessels per section) = 3 and severe (many affected vessels per section) = 5.

	CC75C	Overall average cortical score for CAA (based on maximum scores for either parenchymal or meningeal CAA for each of the four cortical areas) converted back to categorical measure (similar to Love et al. 2014). Severity of vascular amyloid deposits in the brain parenchyma/meninges of frontal, temporal, parietal, and occipital cortices respectively. The pathology is graded as none = 0, sparse (one or two affected vessels per section) =1, moderate (several vessels per section) = 3 and severe (many affected vessels per section) = 5.
	Framingham	Assessment addressed the question: Is (Cerebral) Amyloid angiopathy present? Scored none, mild, moderate, and severe
	HAAS	Neocortical CAA rating: CAA grades were assigned using a system based on the number of CAA-positive parenchymal vessels per area of neocortex.
Diffuse Plaques	RUSH	Semi-quantitative scores (0-5 range) were generated in the frontal cortex, superior temporal cortex, entorhinal cortex, hippocampus CA1, and inferior parietal cortex. Scores of 0 were labeled none, a score of 1-3 was labeled mild, a score of 4 was labeled moderate, and a score of 5 was labeled severe.
	ACT	Variable not available.
	CFAS	By standardized methods (Mirra et al., 1991). Maximum cortical diffuse plaque score. Based on severity of amyloid beta protein-reactive plaque deposits in the frontal, temporal, parietal and occipital cortices respectively. The pathology is graded as none = 0, sparse (one or deposits per section) = 1, moderate (several deposits per section) = 2 and severe (many deposits per section) = 3. Plaque density is referenced to images in CERAD Handbook.
	CC75C	By standardized methods (Mirra et al., 1991). Maximum cortical diffuse plaque score. Based on severity of amyloid beta protein-reactive plaque deposits in the frontal, temporal, parietal and occipital cortices respectively. The pathology is graded as none = 0, sparse (one or deposits per section) = 1, moderate (several deposits per section) = 2 and severe (many deposits per section) = 3. Plaque density is referenced to images in CERAD Handbook.
	Framingham	CERAD Diffuse plaques (plaques with non-compact amyloid and no apparent dystrophic neurites) (through 2014). CERAD semi-quantitative score for diffuse plaques. Score from the neocortical field with the highest plaque density (since 2015)
	HAAS	Variable not available.
Hippocampal Sclerosis	RUSH	Evaluated unilaterally in a coronal section of the mid-hippocampus at the level of the lateral geniculate body. Graded as either absent or present based on severe neuronal loss and gliosis in either the CA1 and/or subiculum. Neuronal loss and gliosis was rated from 0-5 in three regions (Mid hippocampus CA1 - proximal, Mid hippocampus CA1 - distal, Mid hippocampus - subiculum), with higher values indicating more severe neuronal loss. Hippocampal sclerosis rated as present if at least one regions is rated 5.
	ACT	Assessed using hematoxin and eosin-stained sections and recorded as unilateral, bilateral, or laterality unknown but defined as present/absent.

	CFAS	Presence/absence of hippocampal sclerosis as defined in Hokkanen et al. 2017. Based on severe neuron loss in CA1 of the hippocampus, specifically "no more than five neurons per fov in over half of the CA1 fov" at 200x magnification, with the severe neuron loss not explained by ischemic events, and no obvious neuron loss in other hippocampal areas.
	CC75C	Presence/absence of hippocampal sclerosis as defined in Hokkanen et al. 2017. Based on severe neuron loss in CA1 of the hippocampus, specifically "no more than five neurons per fov in over half of the CA1 fov" at 200x magnification, with the severe neuron loss not explained by ischemic events, and no obvious neuron loss in other hippocampal areas.
	Framingham	Assessment of Hippocampal Sclerosis in the CA1 and/or subiculum/ Prosubiculum fields
	HAAS	Hippocampal sclerosis was defined according to conventional neuropathologic criteria as marked neuronal loss and gliosis having sharp margins and being limited to specific parts of the hippocampus.
Lacunes	RUSH	Presence of chronic infarcts in subcortical grey or white matter that are less than or equal to 1 cm in the greatest dimmension.
	ACT	Not assessed.
	CFAS	Presence of lacunes, also labelled as cystic infarcts less than 10mm in diameter assessed grossly.
	CC75C	Presence of lacunes, also labelled as cystic infarcts less than 10mm in diameter assessed grossly.
	Framingham	Acute lacunes in the frontal cortex, parietal cortex, temporal cortex, occipital cortex, subcortical white matter (frontal, parietal, temporal, occipital), caudate, putamen, globus pallidus, internal capsule, thalamus, midbrain, pons, medulla, cerebellum.
Lewy Body Disease	RUSH	Modified McKeith criteria (McKeith et al. 1996) were used to assess Lewy bodies. Individuals with nigral-predominant, limbic-type, or neocortical type Lewy body disease were classified as having Lewy bodies.
	ACT	Modified McKeith criteria (McKeith et al. 1996) were used to assess Lewy bodies. Individuals with brainstem-predominant, limbic (transitional), neocortical (diffuse), or Lewy bodies in the olfactory bulb were classified as having Lewy bodies. Those with amygdala predominant Lewy bodies were classified as not having Lewy bodies.
	CFAS	Summary variable initially operationalised according to McKeith staging: presence/absence of Lewy bodies in brainstem, limbic and cortical areas. This was then dichotomised into an overall presence/absence of Lewy bodies. Based on presence or absence of alpha synuclein reactive Lewy bodies in brainstem (substantia nigra and locus coeruleus); limbic (hippocampus and entorhinal cortex); and cortical areas (frontal, temporal, parietal and occipital cortices). The pathology is graded as none = 0, sparse (one or two affected neurons per section) = 1, moderate (several affected neurons per section) = 2 and severe (many affected neurons per section) = 3. Hematoxylin-eosin or ubiquitin immunohistochemistry was used to visualise Lewy bodies.

	CC75C	Summary variable initially operationalised according to McKeith staging: presence/absence of Lewy bodies in brainstem, limbic and cortical areas. This was then dichotomised into an overall presence/absence of Lewy bodies. Based on presence or absence of alpha synuclein reactive Lewy bodies in brainstem (substantia nigra and locus coeruleus); limbic (hippocampus and entorhinal cortex); and cortical areas (frontal, temporal, parietal and occipital cortices). The pathology is graded as none = 0, sparse (one or two affected neurons per section) = 1, moderate (several affected neurons per section) = 2 and severe (many affected neurons per section) = 3. Initial donors used hematoxylin-eosin or ubiquitin immunohistochemistry, later donors used alpha synuclein immunohistochemistry.
	Framingham	Assessment of Lewy body pathology consistent with criteria of Consortium on Dementia with Lewy Bodies for brainstem predominant, limbic (transitional), neocortical (diffuse), olfactory bulb were classified as having Lewy bodies
	HAAS	Based on a modified McKeith criteria (McKeith et al. 1996). Individuals with brainstem predominant, limbic predominant, or neocortical predominant Lewy body dementia were classified as having Lewy bodies.
Macroinfarcts	RUSH	Presence/absence of chronic infarcts assessed in at least nine regions (the midfrontal, middle temporal, entorhinal, hippocampal, inferior parietal, and anterior cingulate cortices, as well as the anterior basal ganglia, thalamus, and midbrain).
	ACT	Old or gross infarcts were defined as present/absent. Lacunes (defined as small infarcts) were included.
	CFAS	Presence of any gross parenchymal lesions - infarcts.
	CC75C	Presence of any gross parenchymal lesions - infarcts.
	Framingham	Chronic gross infarcts (>1cm in diameter) including lacunes in the fronto-polar, orbito-frontal, middle-frontal, superior-frontal, anterior cingulate, rolandic, posterior-medial frontal, broca, temporo-polar, inferior temporal, amygdala, hippocampus, middle temporal, superior temporal, auditory area, inferior parietal, superior parietal, angular gyrus, calcarine, inferior/lateral occipital, basal forebrain, caudate, putamen, global pallidus, internal capsule, thalamus, midbrain, pons, medulla, cerebellum, MCA -, ACA -, PCA-, ACA-/MCA- territories were included
	HAAS	Infarcts whose longest dimmension exceeded 1 cm.
Microinfarcts	RUSH	Presence/absence of chronic microinfarcts assessed in at least nine regions (the midfrontal, middle temporal, entorhinal, hippocampal, inferior parietal, and anterior cingulate cortices, as well as the anterior basal ganglia, thalamus, and midbrain).
	ACT	Presence of any chronic infarcts observed microscopically (i.e., NOT observed grossly) in 'cerebral' and 'deep' screening sections. 'Cerebral' includes: middle frontal gyrus, middle superior temporal gyrus, inferior parietal lobule, and occipital lobe. 'Deep' includes striatum and thalamus.
	CFAS	Presence or absence of microinfarcts.
	CC75C	Presence or absence of microinfarcts.
	Framingham	Assessment addressed the question: Are one or more cortical, microinfarcts (including “granular atrophy”) present?

	HAAS	Microinfarcts were defined as temporally remote (judged older than 1 month) microscopic (not seen at gross examination) foci of neuronal loss and gliosis, or of focal leukoencephalopathy when the lesion occurred in white matter. Total microinfarcts were typically counted for several brain regions on standard H&E section representing 0.5–1.5 square cm of tissue. The microinfarct counts used for these analyses were the total number identified on sections from the isocortex (8 sections), caudate (n = 2), putamen (n = 2), globus pallidus (n = 2), thalamus (n = 2), hippocampus (n = 2), nucleus basalis (n = 2), amygdala (n = 2), brainstem (n = 1), pons (n = 1), and cerebellum (n = 2).
LATE-NC	RUSH	TDP-43 immunohistochemistry performed on 8 regions (amygdala, entorhinal cortex, hippocampus CA1, hippocampus dentate gyrus, anterior temporal pole cortex, midtemporal cortex, orbital frontal cortex, midfrontal cortex) using phosphorylated monoclonal TAR5P-1D3. No LATE-NC included those with either no TDP-43 or TDP-43 in the amygdala. Presence of LATE-NC corresponded to TDP-43 inclusions in the limbic or neocortical regions (LATE-NC stage >=2).
	ACT	Presence/absence of LATE-NC stage >=2 (staging as recommended by Nelson et al. 2019).
	CFAS	Presence/absence of LATE-NC stage >=2 (staging as recommended by Nelson et al. 2019). Based on assessment of TDP-43 proteinopathy in the hippocampus and parahippocampal gyrus, with any score greater than zero denoting presence of TDP-43 proteinopathy in that area.
	CC75C	Presence/absence of LATE-NC stage >=2 (staging as recommended by Nelson et al. 2019). Based on assessment of TDP-43 proteinopathy in the hippocampus and parahippocampal gyrus, with any score greater than zero denoting presence of TDP-43 proteinopathy in that area.
	Framingham	No FTLD with TDP-43 pathology, and presence of TDP-43 in the Amygdala, Hippocampus, Entorhinal/inferior temporal cortex, and Neocortex regions (measures available since 2015)
	HAAS	Not assessed.

Appendix 5: Meta-analyses of polychoric correlations to summarize across cohorts

Appendix Figure S3. Random-effects meta-analyses of polychoric correlations calculated in each cohort separately were used to derive summary measures of correlations between each pair of neuropathology measures

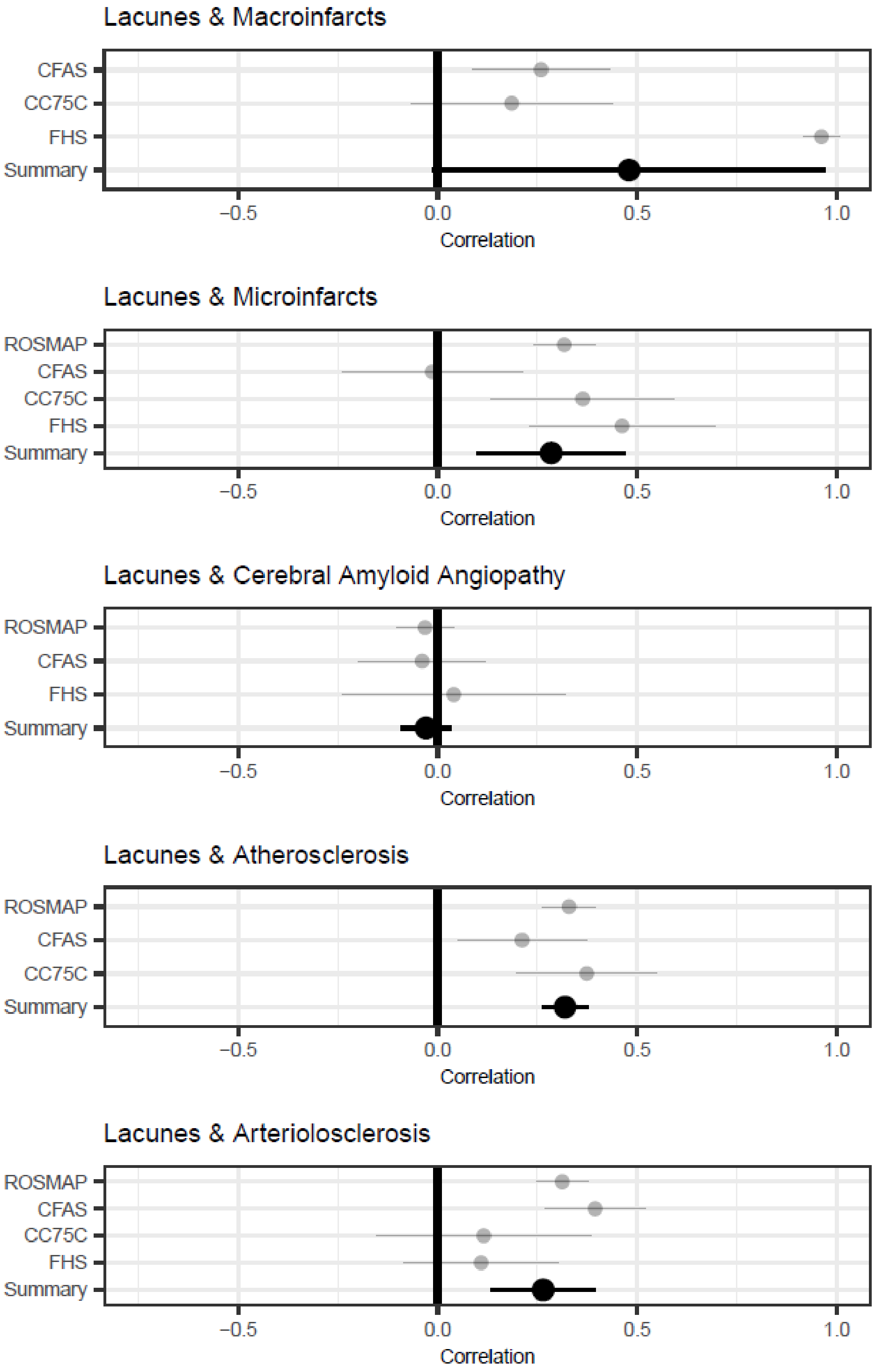

Lacunes & Hippocampal Sclerosis

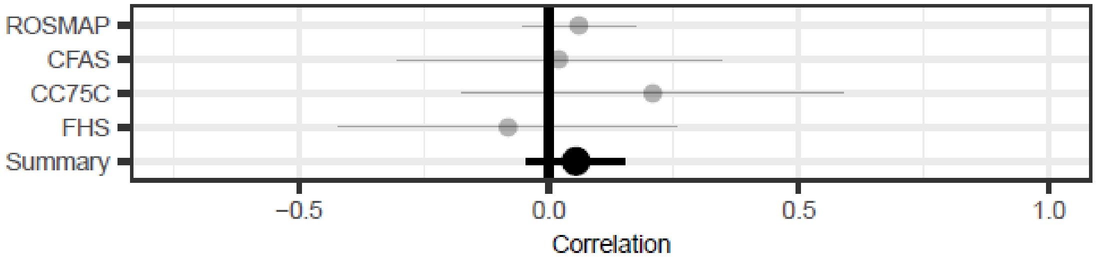

Lacunes & Lewy Body Disease

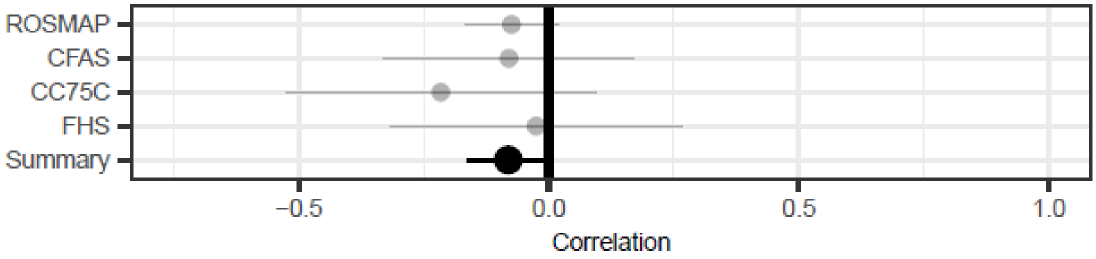

Lacunes & CERAD Neuritic Plaque Score

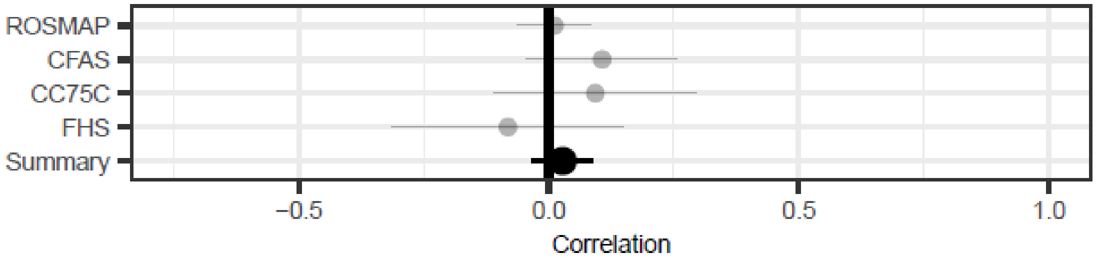

Lacunes & CERAD Diffuse Plaque Score

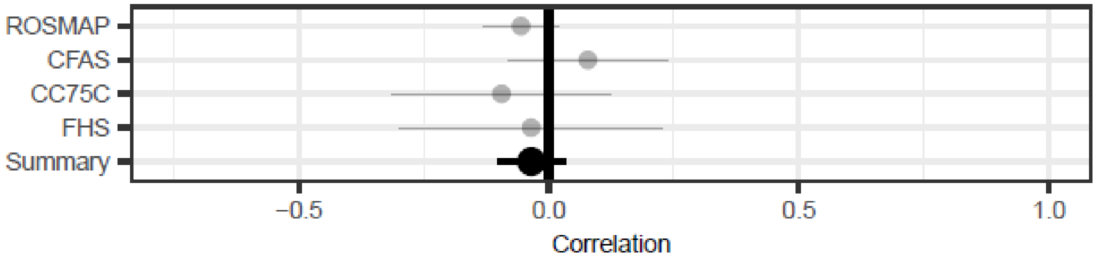

Lacunes & Braak Score

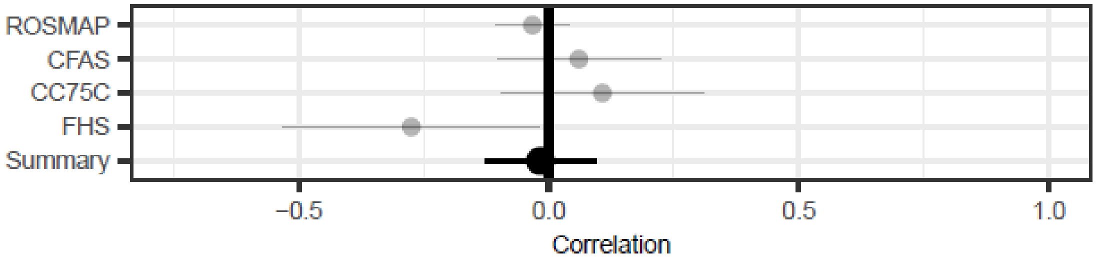

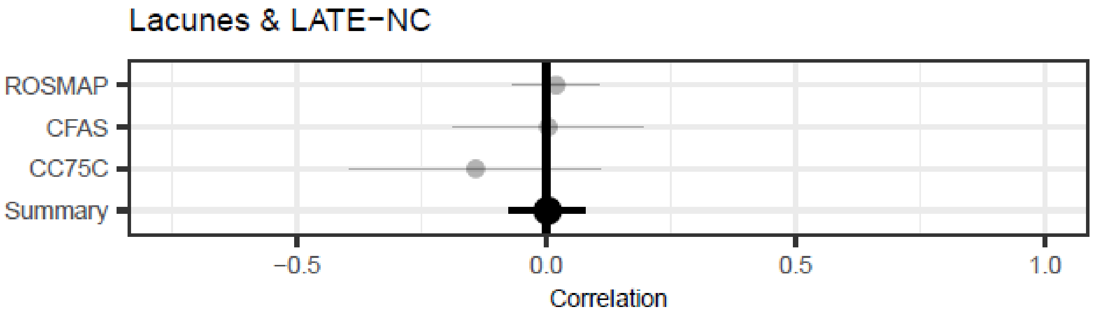
Lacunes & LATE−NC
ROSMAP
CFAS
CC75C
Summary
−0.5
0.0
0.5
1.0
Correlation

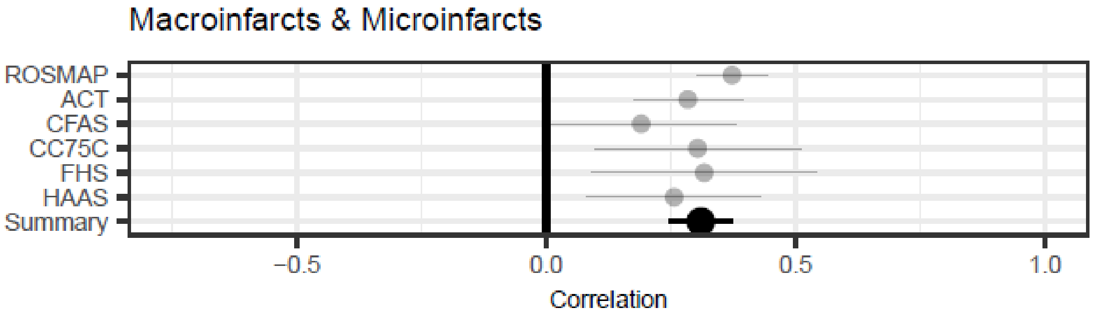
Macroinfarcts & Microinfarcts
ROSMAP
ACT
CFAS
CC75C
FHS
HAAS
Summary
−0.5
0.0
0.5
1.0
Correlation

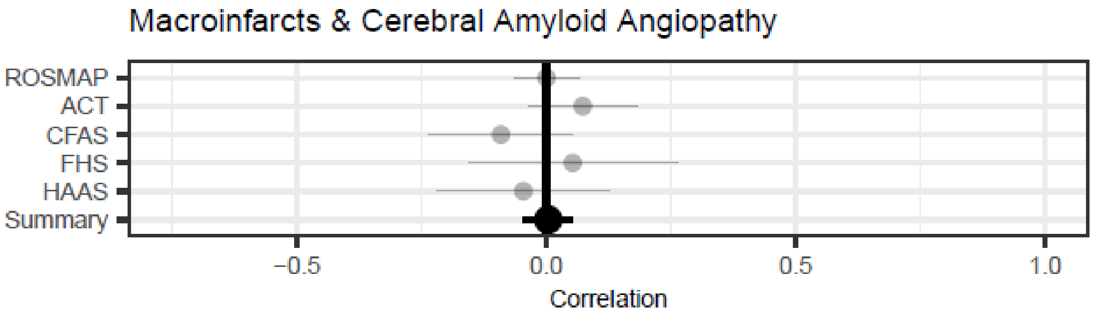
Macroinfarcts & Cerebral Amyloid Angiopathy
ROSMAP
ACT
CFAS
FHS
HAAS
Summary
−0.5
0.0
0.5
1.0
Correlation

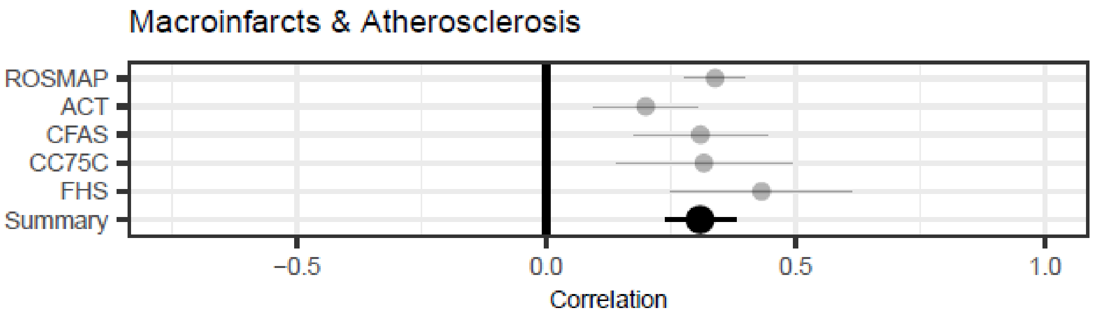
Macroinfarcts & Atherosclerosis
ROSMAP
ACT
CFAS
CC75C
FHS
Summary
−0.5
0.0
0.5
1.0
Correlation

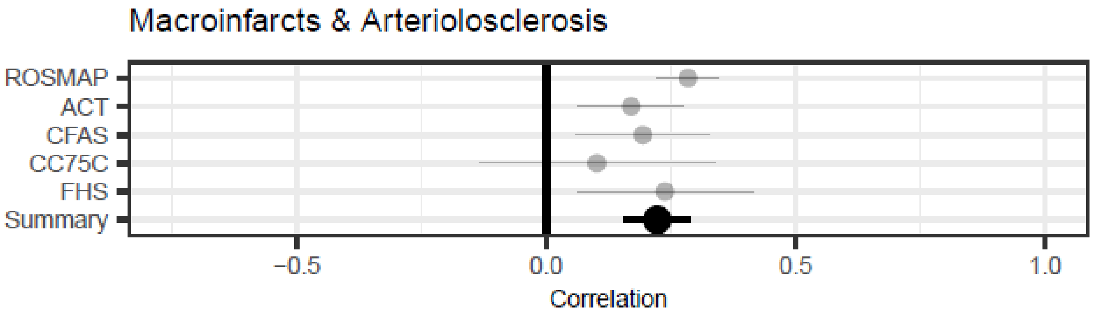
Macroinfarcts & Arteriolosclerosis
ROSMAP
ACT
CFAS
CC75C
FHS
Summary
−0.5
0.0
0.5
1.0
Correlation

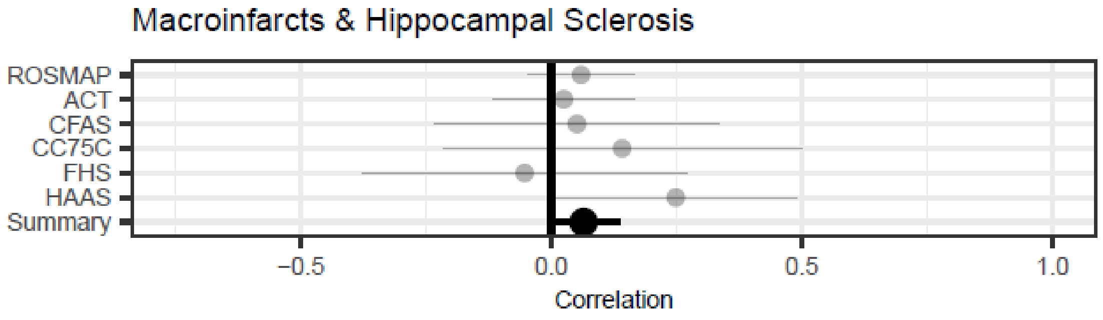
Macroinfarcts & Hippocampal Sclerosis
ROSMAP
ACT
CFAS
CC75C
FHS
HAAS
Summary
−0.5
0.0
0.5
1.0
Correlation

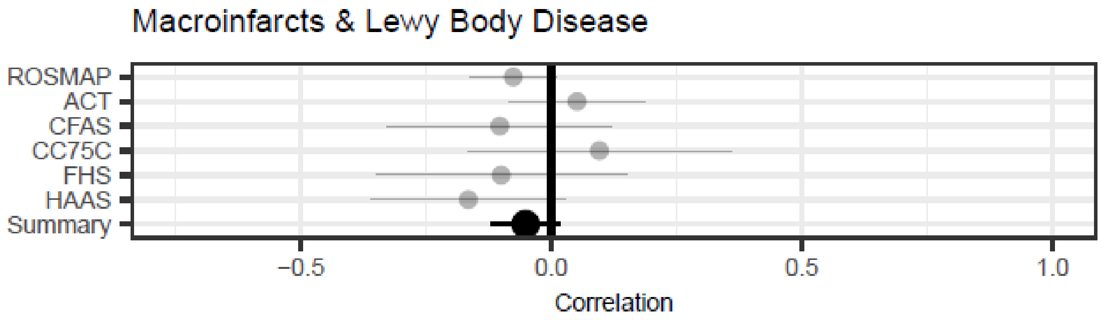
Macroinfarcts & Lewy Body Disease
ROSMAP
ACT
CFAS
CC75C
FHS
HAAS
Summary
−0.5
0.0
0.5
1.0
Correlation

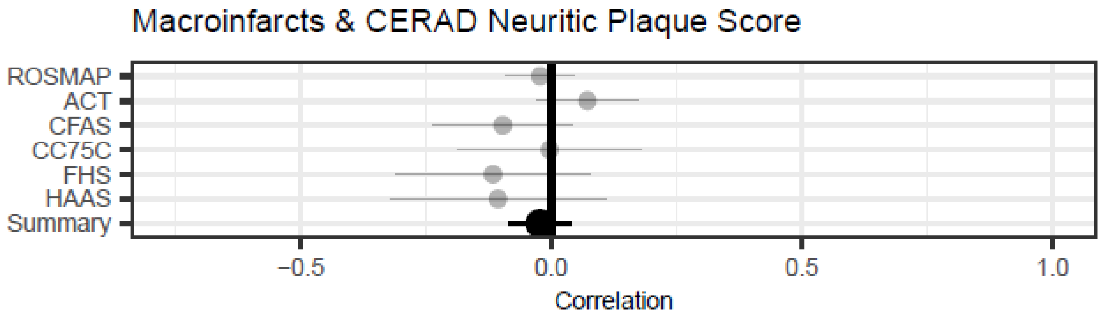
Macroinfarcts & CERAD Neuritic Plaque Score
ROSMAP
ACT
CFAS
CC75C
FHS
HAAS
Summary
−0.5
0.0
0.5
1.0
Correlation

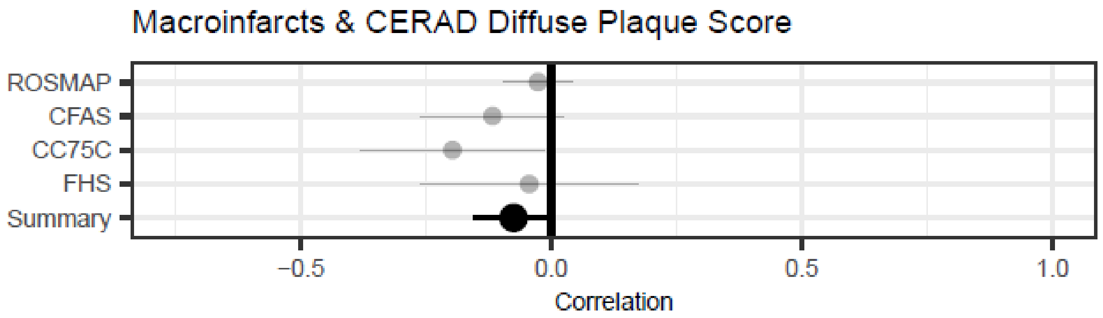
Macroinfarcts & CERAD Diffuse Plaque Score
ROSMAP
CFAS
CC75C
FHS
Summary
−0.5
0.0
0.5
1.0
Correlation

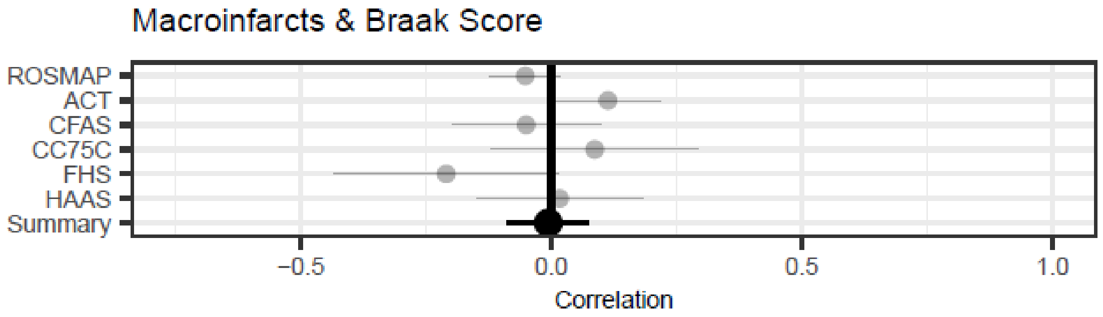
Macroinfarcts & Braak Score
ROSMAP
ACT
CFAS
CC75C
FHS
HAAS
Summary
−0.5
0.0
0.5
1.0
Correlation

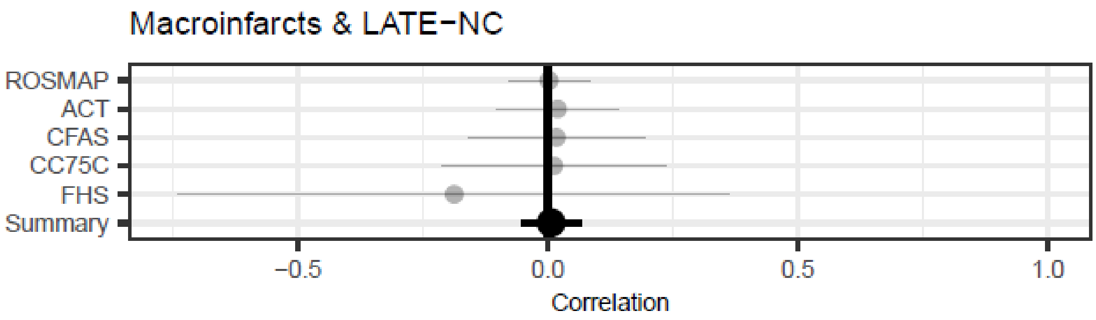
Macroinfarcts & LATE−NC
ROSMAP
ACT
CFAS
CC75C
FHS
Summary
−0.5
0.0
0.5
1.0
Correlation

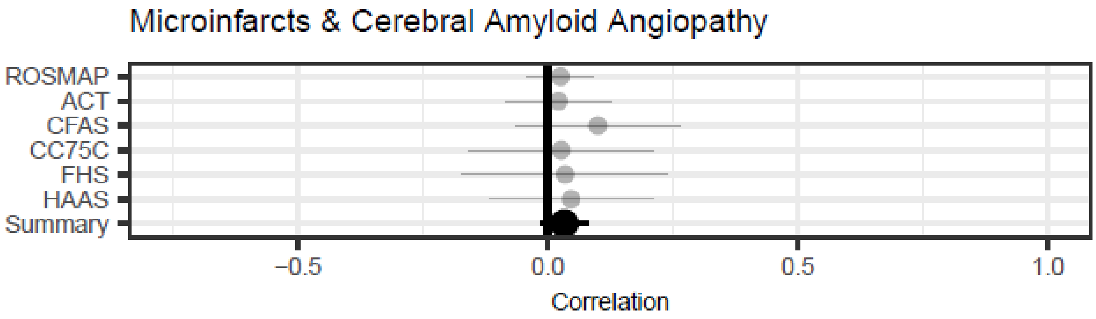
Microinfarcts & Cerebral Amyloid Angiopathy
ROSMAP
ACT
CFAS
CC75C
FHS
HAAS
Summary
−0.5
0.0
0.5
1.0
Correlation

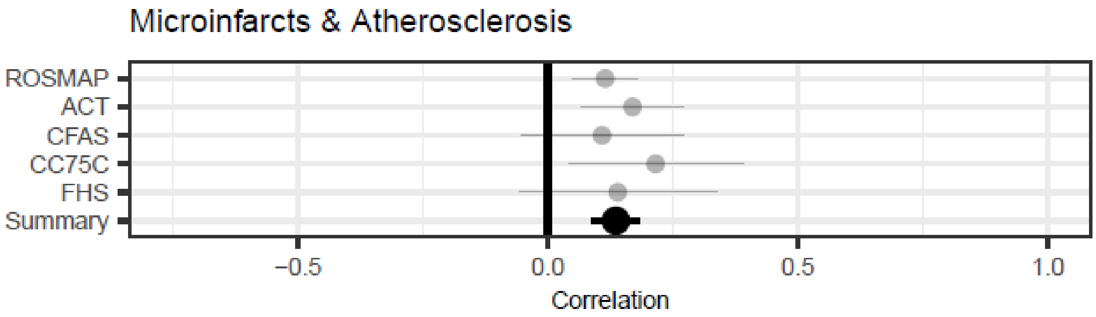
Microinfarcts & Atherosclerosis
ROSMAP
ACT
CFAS
CC75C
FHS
Summary
−0.5
0.0
0.5
1.0
Correlation

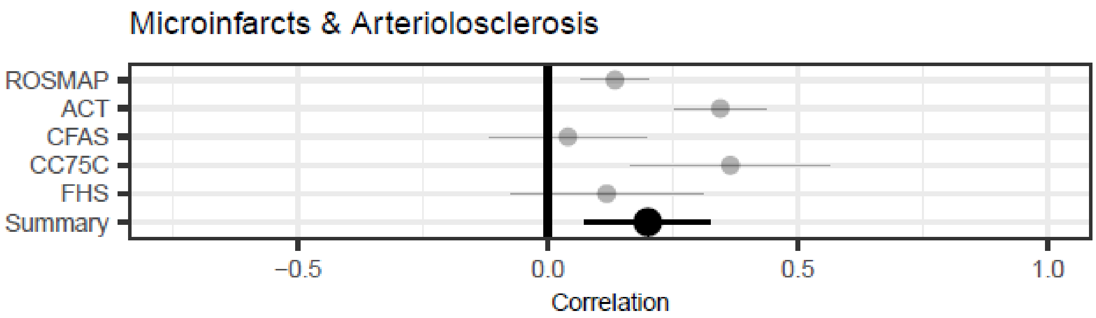
Microinfarcts & Arteriolosclerosis
ROSMAP
ACT
CFAS
CC75C
FHS
Summary
−0.5
0.0
0.5
1.0
Correlation

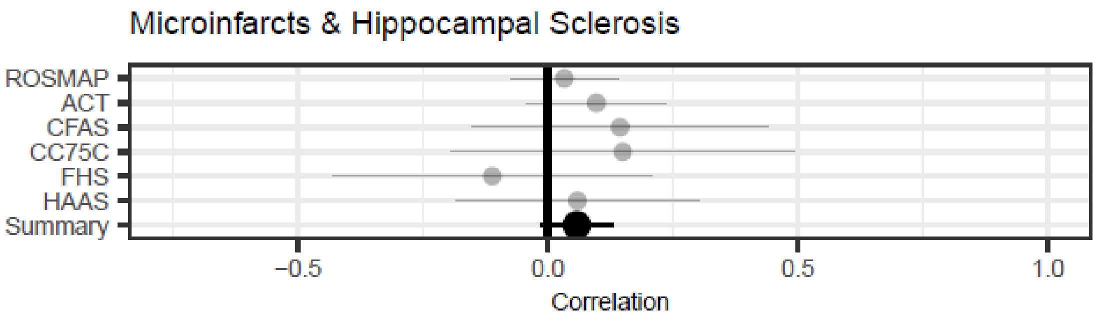
Microinfarcts & Hippocampal Sclerosis
ROSMAP
ACT
CFAS
CC75C
FHS
HAAS
Summary
−0.5
0.0
0.5
1.0
Correlation

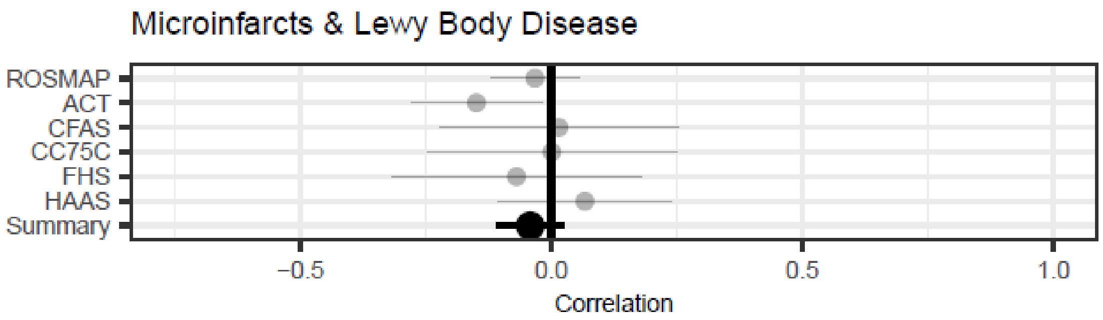
Microinfarcts & Lewy Body Disease
ROSMAP
ACT
CFAS
CC75C
FHS
HAAS
Summary
−0.5
0.0
0.5
1.0
Correlation

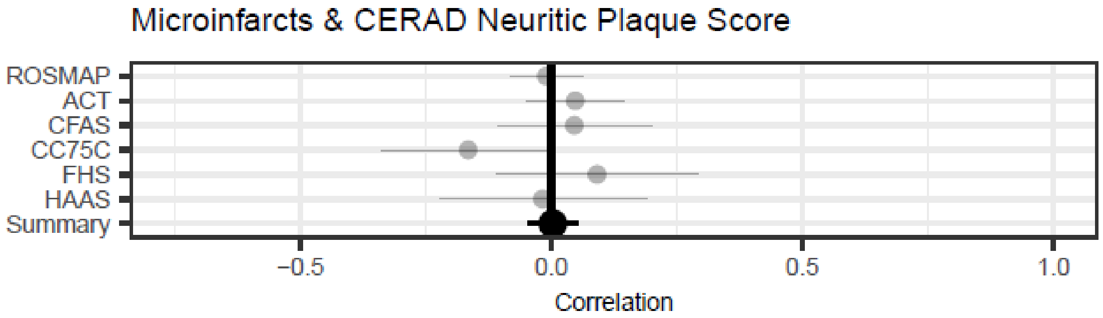
Microinfarcts & CERAD Neuritic Plaque Score
ROSMAP
ACT
CFAS
CC75C
FHS
HAAS
Summary
−0.5
0.0
0.5
1.0
Correlation

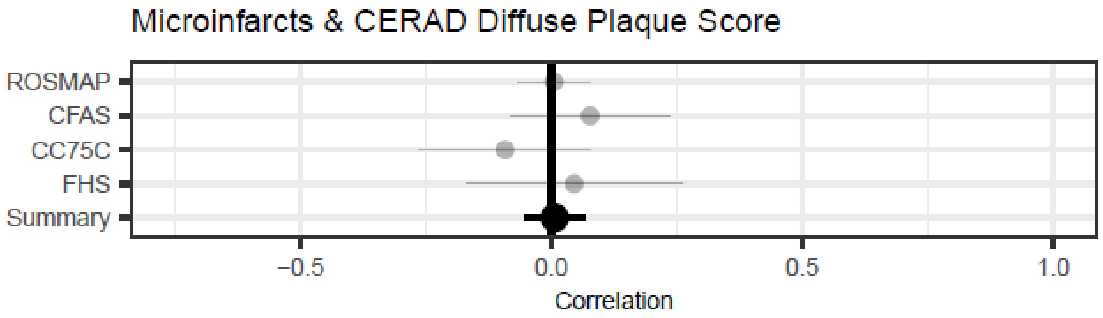
Microinfarcts & CERAD Diffuse Plaque Score
ROSMAP
CFAS
CC75C
FHS
Summary
−0.5
0.0
0.5
1.0
Correlation

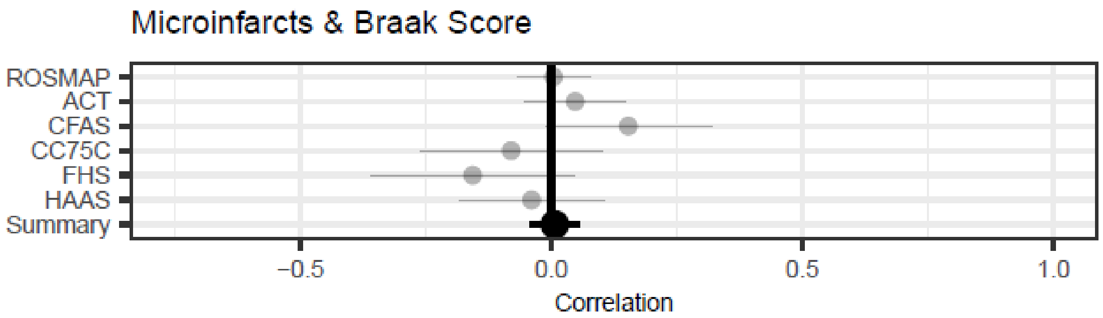
Microinfarcts & Braak Score
ROSMAP
ACT
CFAS
CC75C
FHS
HAAS
Summary
−0.5
0.0
0.5
1.0
Correlation

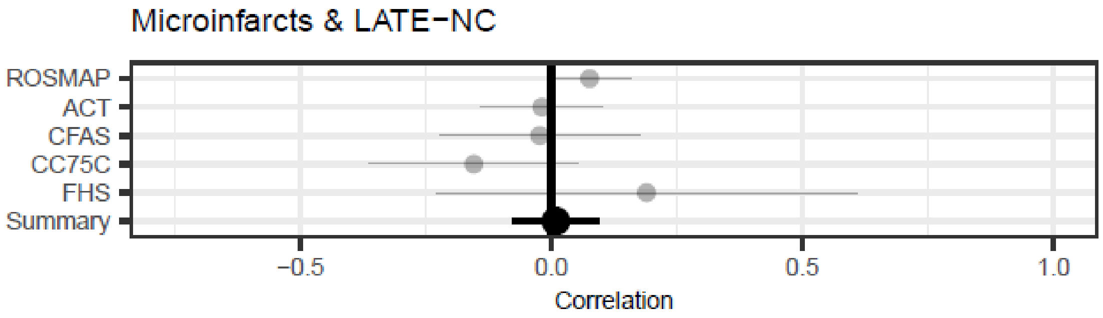
Microinfarcts & LATE−NC
ROSMAP
ACT
CFAS
CC75C
FHS
Summary
−0.5
0.0
0.5
1.0
Correlation

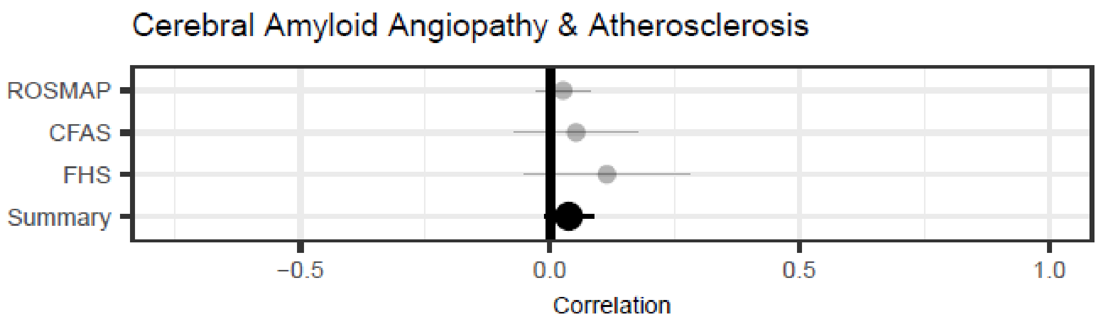
Cerebral Amyloid Angiopathy & Atherosclerosis
ROSMAP
CFAS
FHS
Summary
−0.5
0.0
0.5
1.0
Correlation

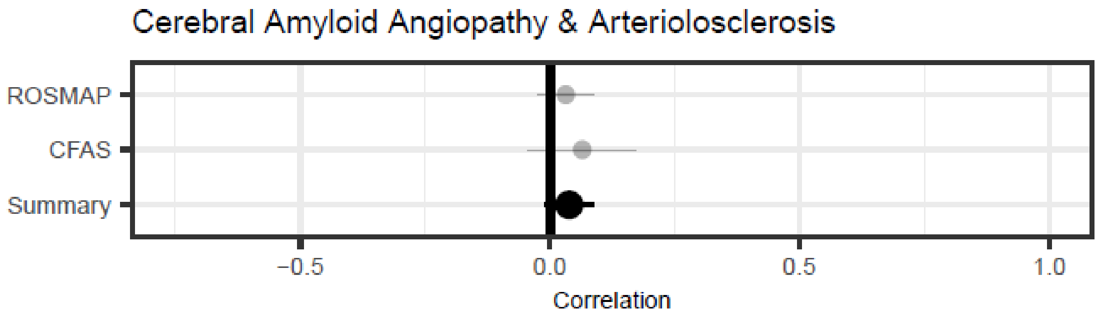
Cerebral Amyloid Angiopathy & Arteriolosclerosis
ROSMAP
CFAS
Summary
−0.5
0.0
0.5
1.0
Correlation

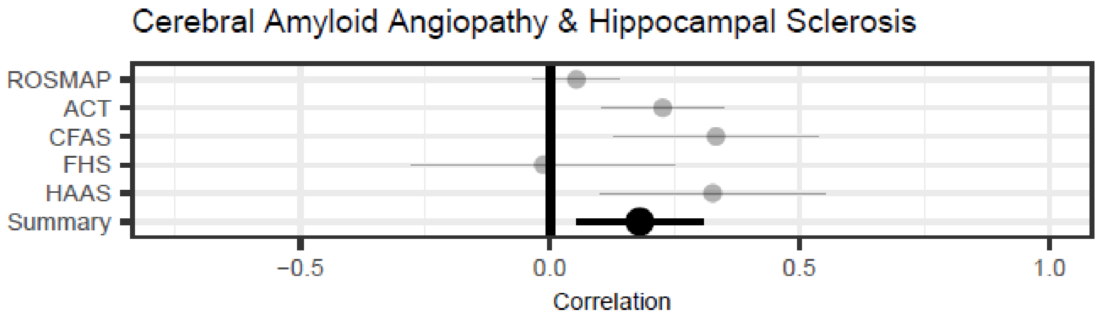
Cerebral Amyloid Angiopathy & Hippocampal Sclerosis
ROSMAP
ACT
CFAS
FHS
HAAS
Summary
−0.5
0.0
0.5
1.0
Correlation

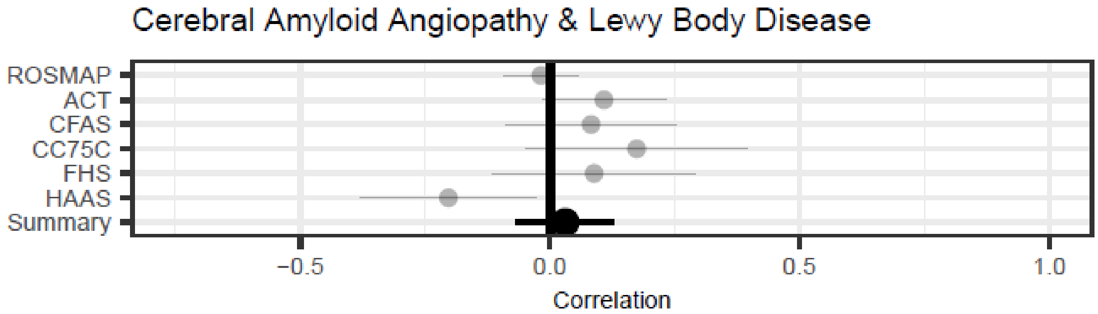
Cerebral Amyloid Angiopathy & Lewy Body Disease
ROSMAP
ACT
CFAS
CC75C
FHS
HAAS
Summary
−0.5
0.0
0.5
1.0
Correlation

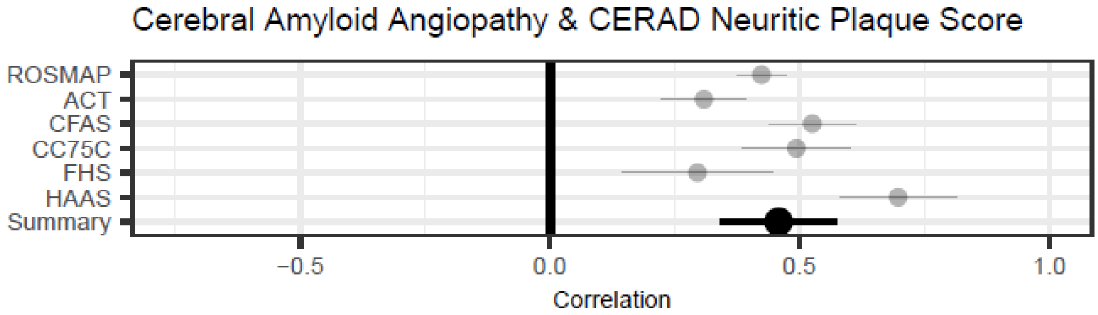
Cerebral Amyloid Angiopathy & CERAD Neuritic Plaque Score
ROSMAP
ACT
CFAS
CC75C
FHS
HAAS
Summary
−0.5
0.0
0.5
1.0
Correlation

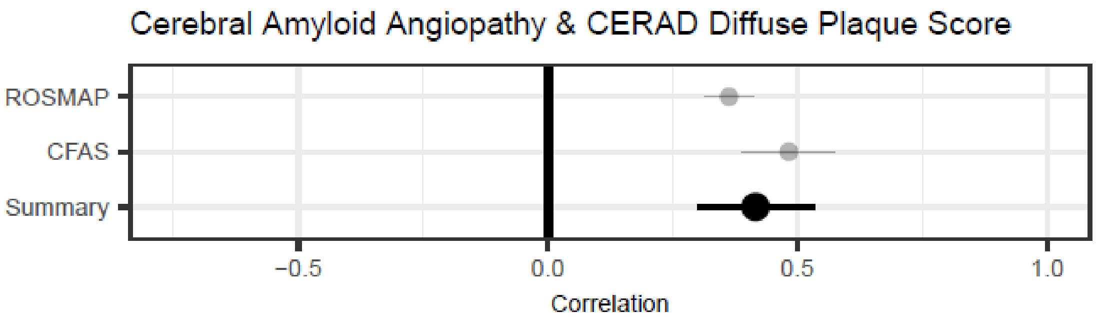
Cerebral Amyloid Angiopathy & CERAD Diffuse Plaque Score
ROSMAP
CFAS
Summary
−0.5
0.0
0.5
1.0
Correlation

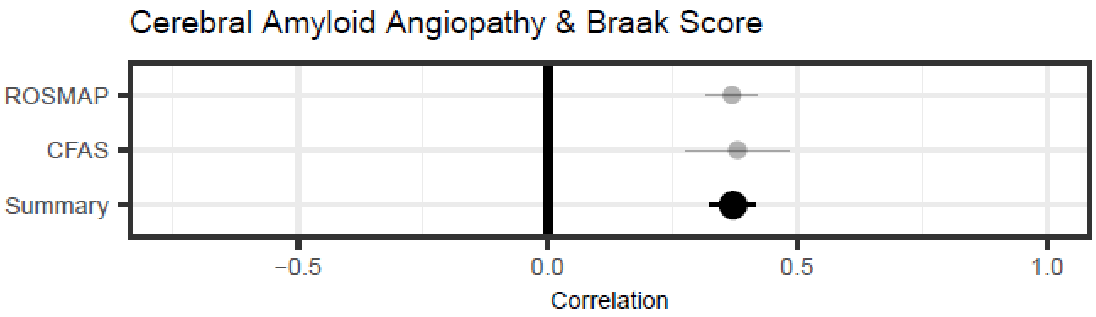
Cerebral Amyloid Angiopathy & Braak Score
ROSMAP
CFAS
Summary
−0.5
0.0
0.5
1.0
Correlation

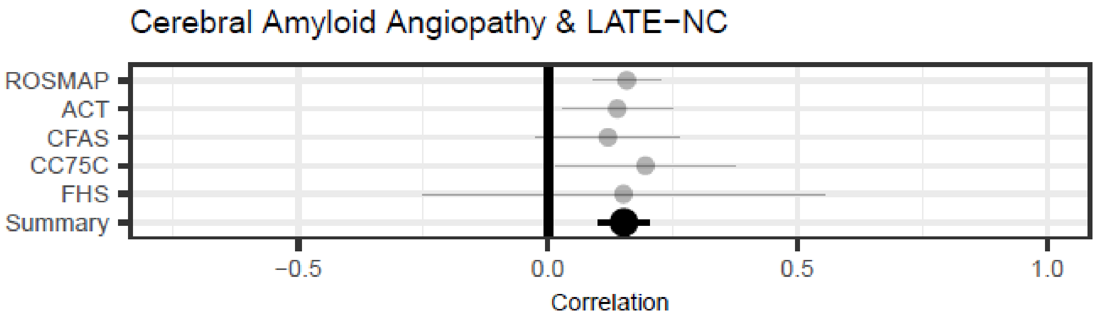
Cerebral Amyloid Angiopathy & LATE−NC
ROSMAP
ACT
CFAS
CC75C
FHS
Summary
−0.5
0.0
0.5
1.0
Correlation

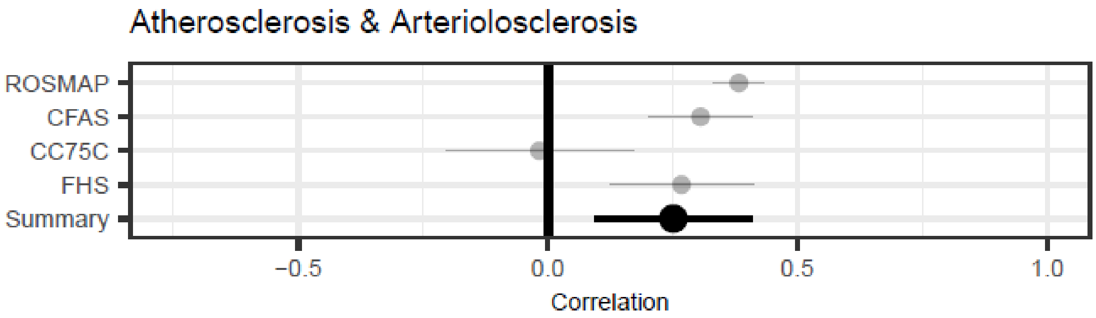
Atherosclerosis & Arteriolosclerosis
ROSMAP
CFAS
CC75C
FHS
Summary
−0.5
0.0
0.5
1.0
Correlation

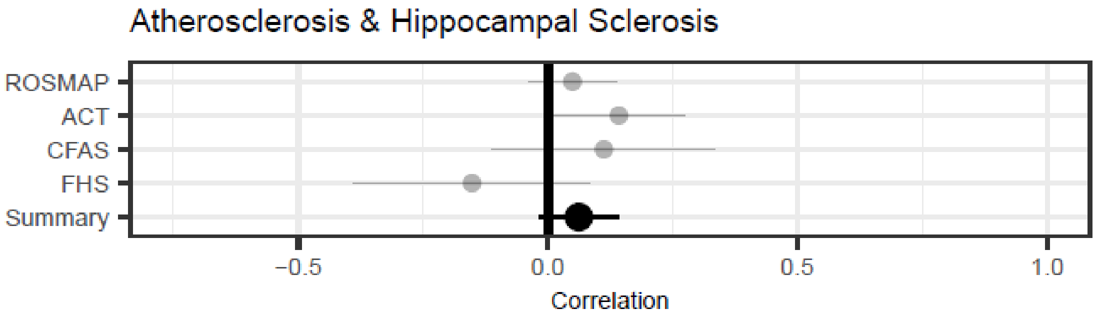
Atherosclerosis & Hippocampal Sclerosis
ROSMAP
ACT
CFAS
FHS
Summary
−0.5
0.0
0.5
1.0
Correlation

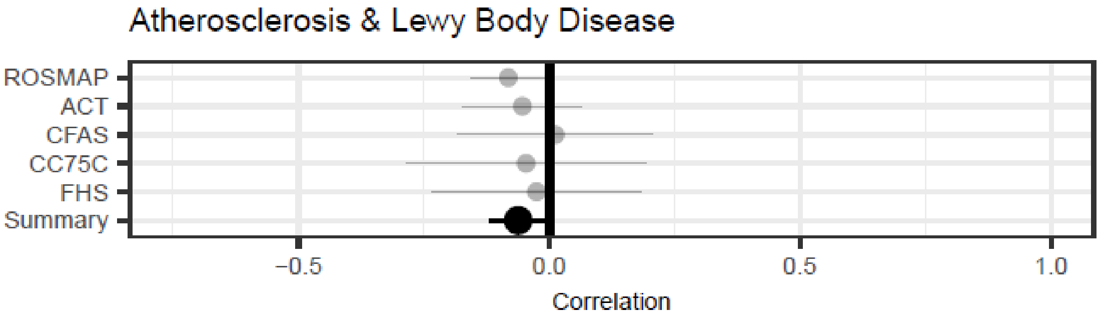
Atherosclerosis & Lewy Body Disease
ROSMAP
ACT
CFAS
CC75C
FHS
Summary
−0.5
0.0
0.5
1.0
Correlation

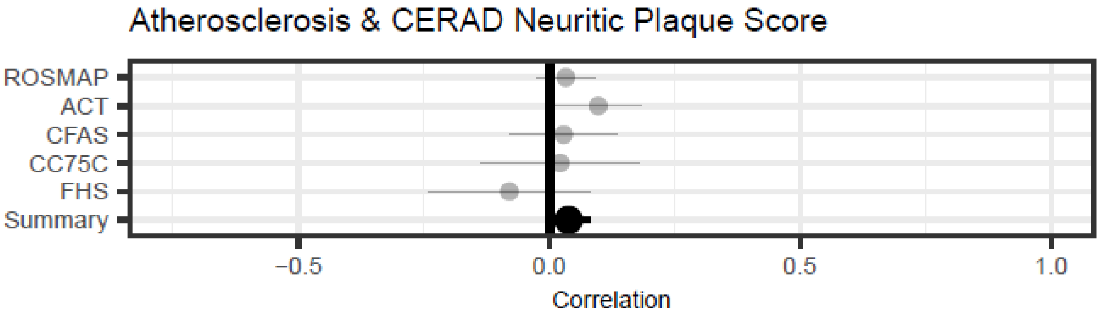
Atherosclerosis & CERAD Neuritic Plaque Score
ROSMAP
ACT
CFAS
CC75C
FHS
Summary
−0.5
0.0
0.5
1.0
Correlation

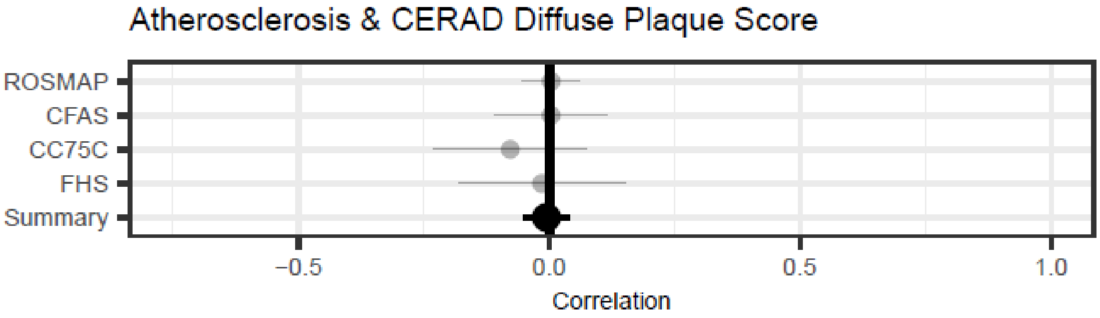
Atherosclerosis & CERAD Diffuse Plaque Score
ROSMAP
CFAS
CC75C
FHS
Summary
−0.5
0.0
0.5
1.0
Correlation

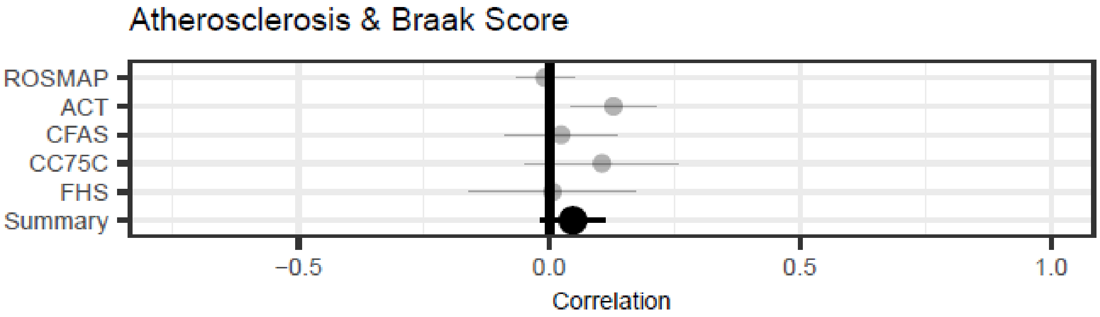
Atherosclerosis & Braak Score
ROSMAP
ACT
CFAS
CC75C
FHS
Summary
−0.5
0.0
0.5
1.0
Correlation

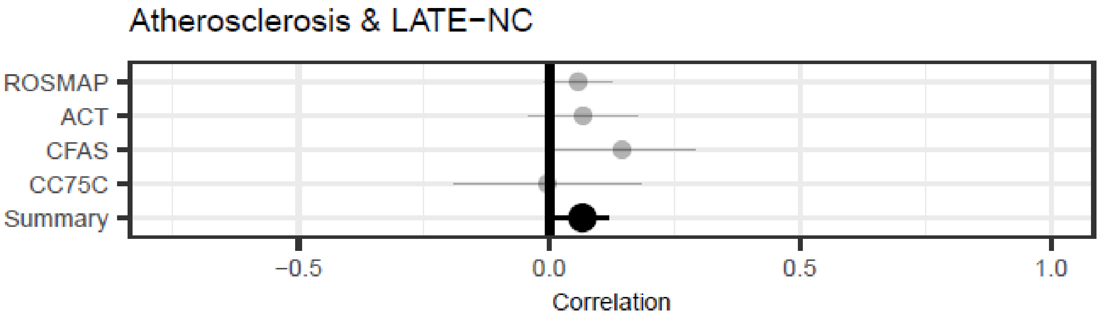
Atherosclerosis & LATE−NC
ROSMAP
ACT
CFAS
CC75C
Summary
−0.5
0.0
0.5
1.0
Correlation

Arteriolosclerosis & Hippocampal Sclerosis

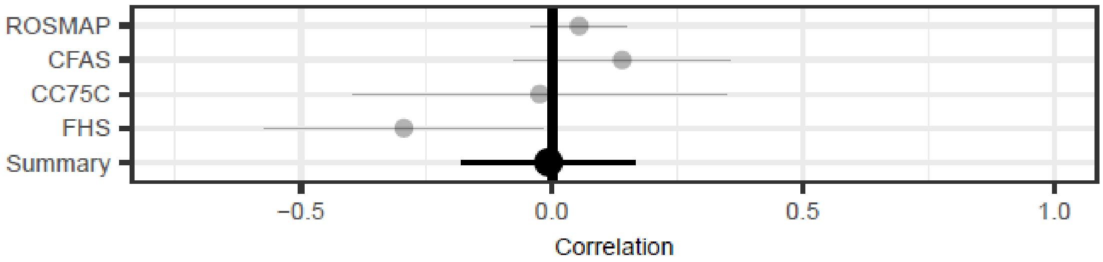

Arteriolosclerosis & Lewy Body Disease

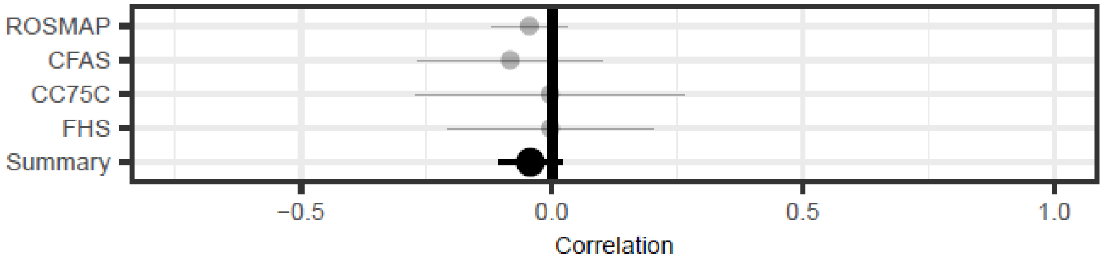

Arteriolosclerosis & CERAD Neuritic Plaque Score

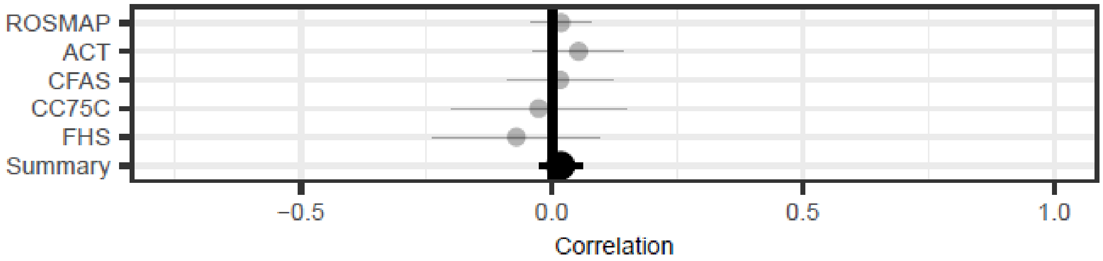

Arteriolosclerosis & CERAD Diffuse Plaque Score

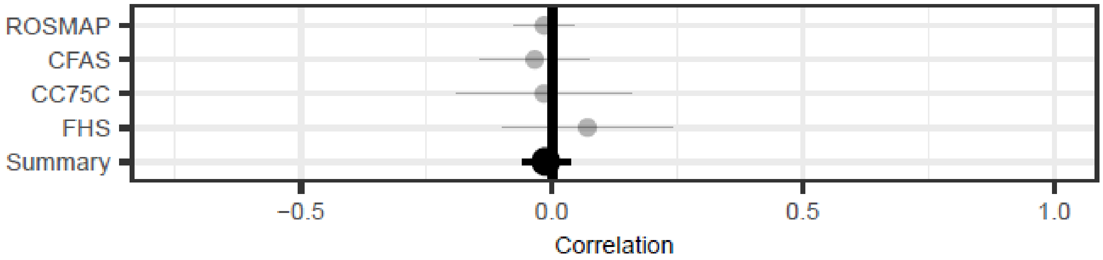

Arteriolosclerosis & Braak Score

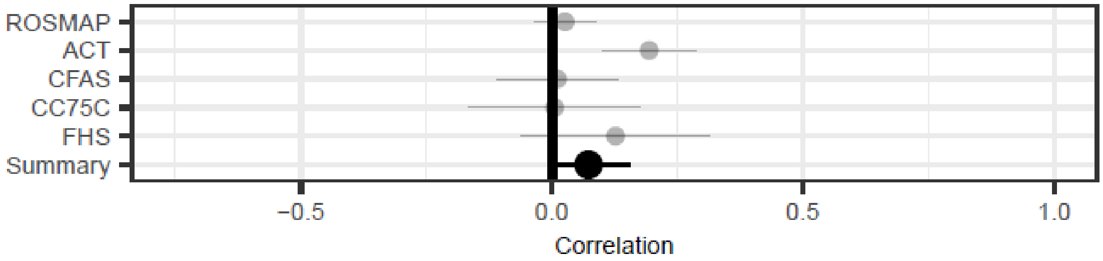

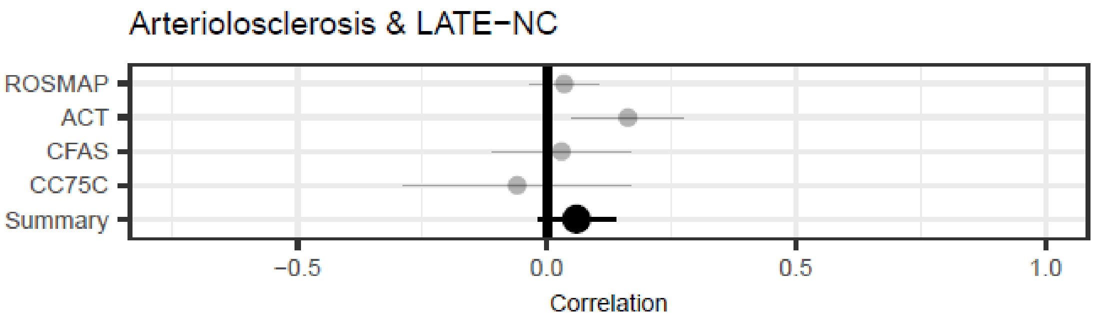
Arteriolosclerosis & LATE−NC
ROSMAP
ACT
CFAS
CC75C
Summary
−0.5
0.0
0.5
1.0
Correlation

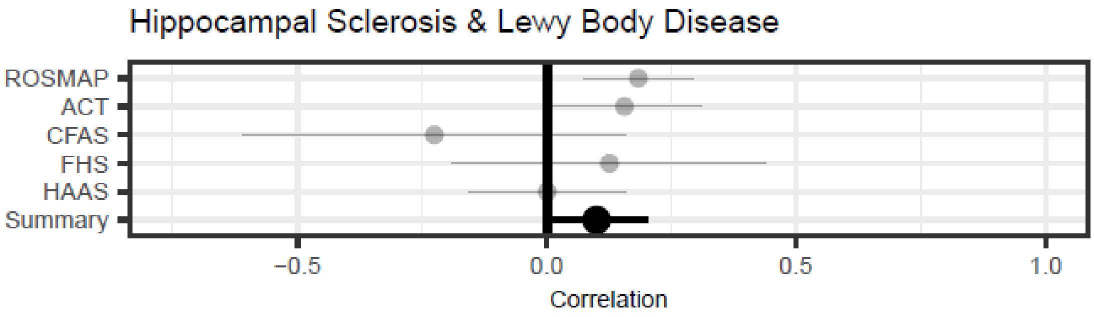
Hippocampal Sclerosis & Lewy Body Disease
ROSMAP
ACT
CFAS
FHS
HAAS
Summary
−0.5
0.0
0.5
1.0
Correlation

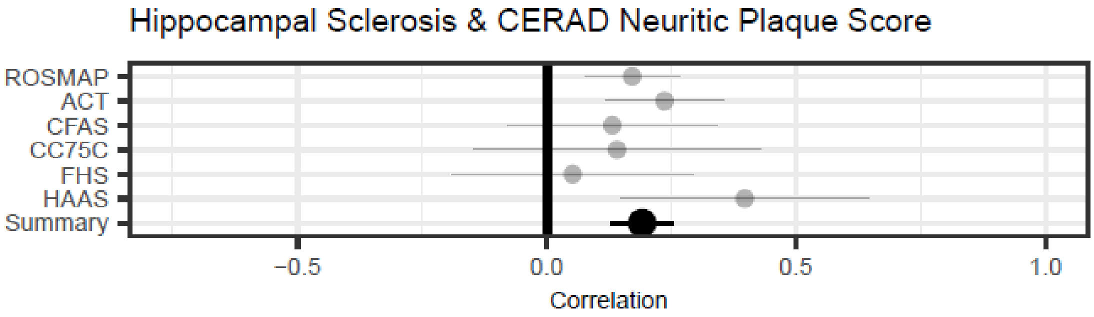
Hippocampal Sclerosis & CERAD Neuritic Plaque Score
ROSMAP
ACT
CFAS
CC75C
FHS
HAAS
Summary
−0.5
0.0
0.5
1.0
Correlation

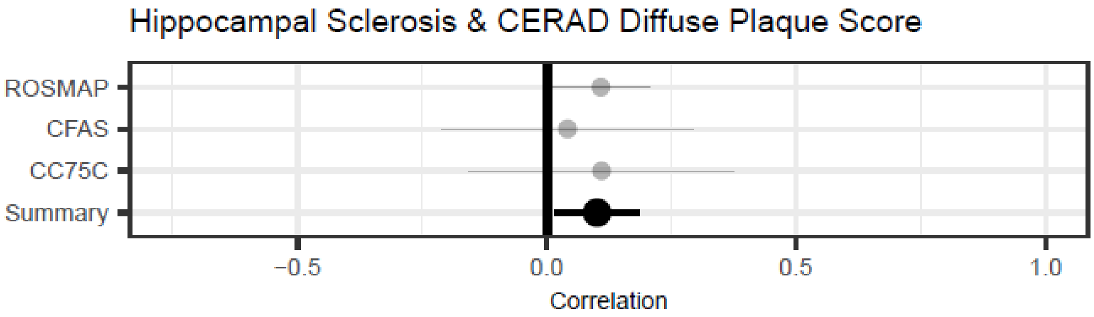
Hippocampal Sclerosis & CERAD Diffuse Plaque Score
ROSMAP
CFAS
CC75C
Summary
−0.5
0.0
0.5
1.0
Correlation

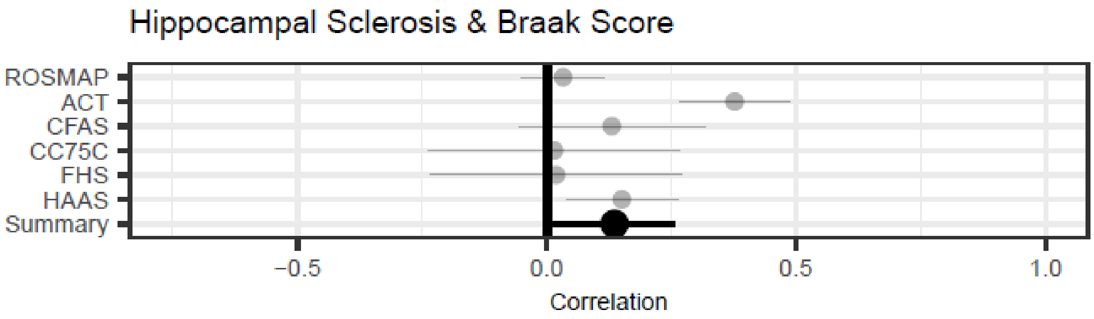
Hippocampal Sclerosis & Braak Score
ROSMAP
ACT
CFAS
CC75C
FHS
HAAS
Summary
−0.5
0.0
0.5
1.0
Correlation

Hippocampal Sclerosis & LATE−NC

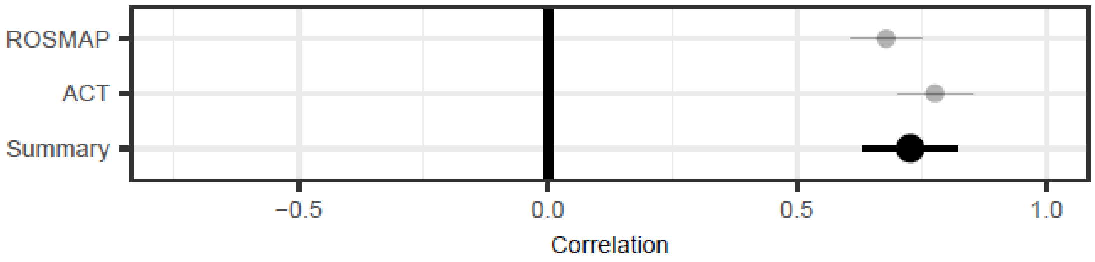

Lewy Body Disease & CERAD Neuritic Plaque Score

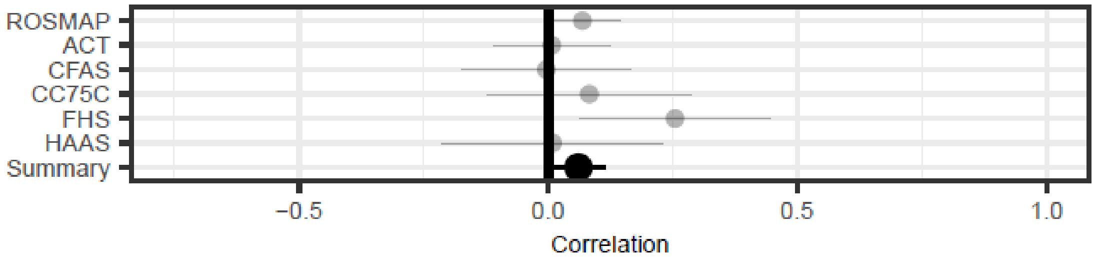

Lewy Body Disease & CERAD Diffuse Plaque Score

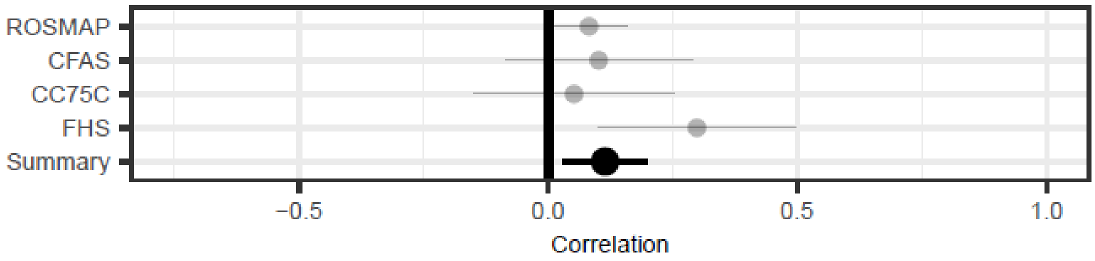

Lewy Body Disease & Braak Score

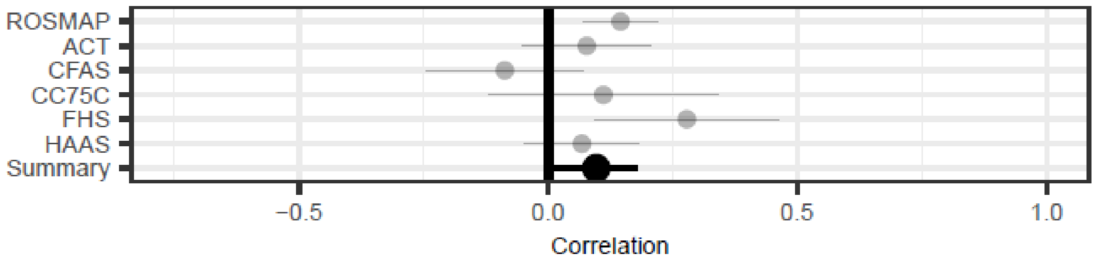

Lewy Body Disease & LATE−NC

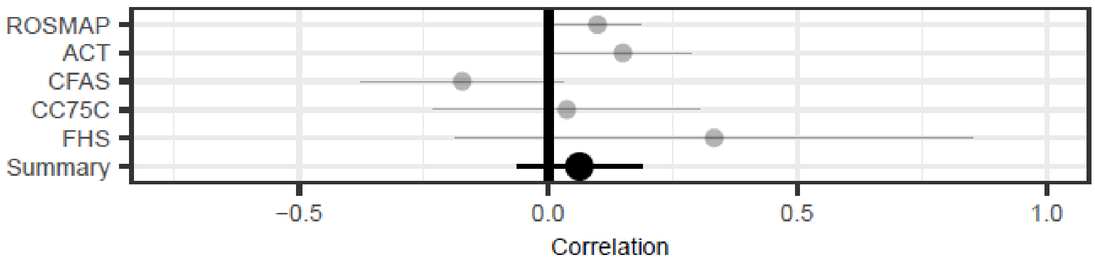

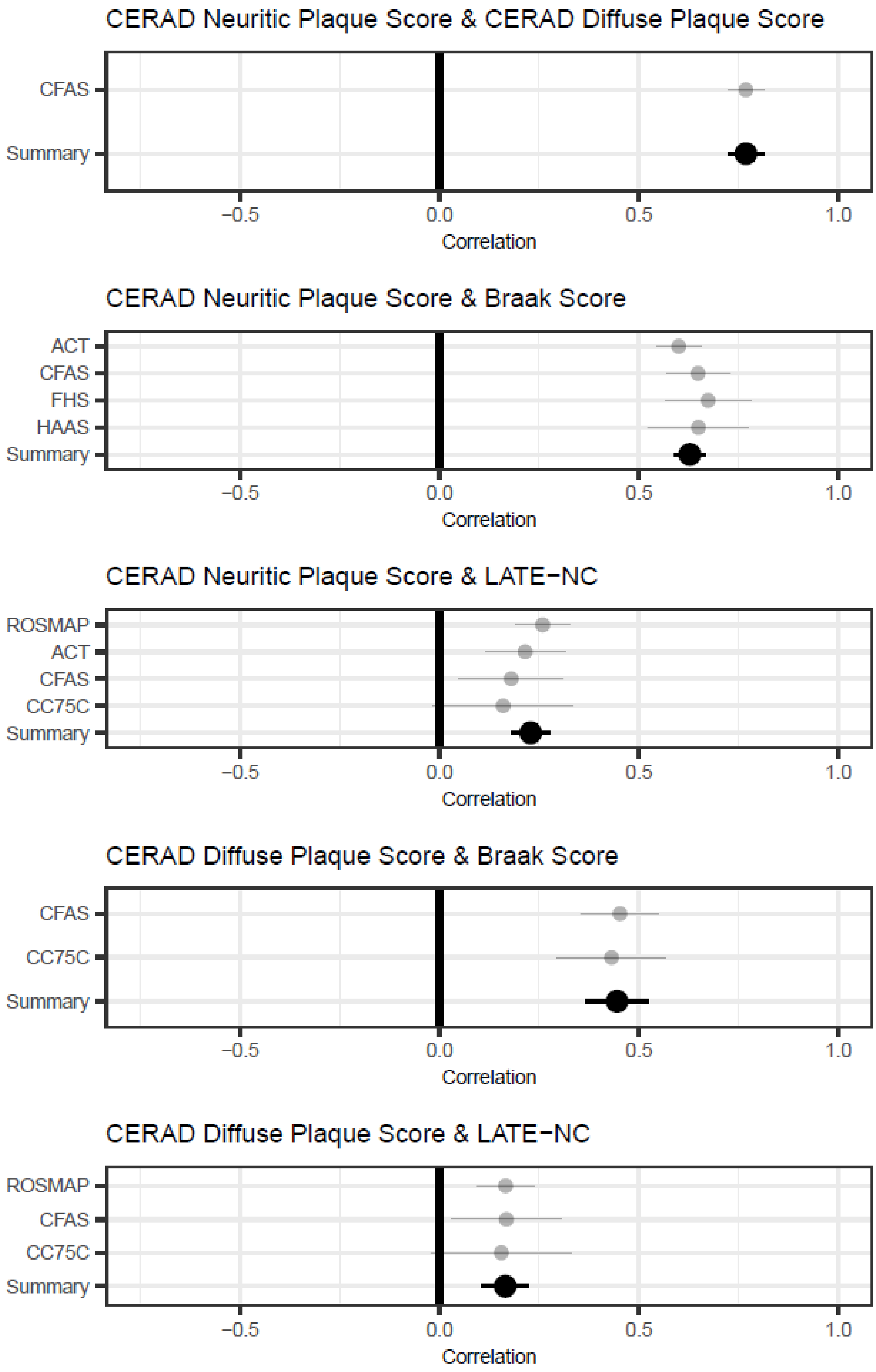
CERAD Neuritic Plaque Score & CERAD Diffuse Plaque Score
CFAS
Summary
−0.5
0.0
0.5
1.0
Correlation
CERAD Neuritic Plaque Score & Braak Score
ACT
CFAS
FHS
HAAS
Summary
−0.5
0.0
0.5
1.0
Correlation
CERAD Neuritic Plaque Score & LATE−NC
ROSMAP
ACT
CFAS
CC75C
Summary
−0.5
0.0
0.5
1.0
Correlation
CERAD Diffuse Plaque Score & Braak Score
CFAS
CC75C
Summary
−0.5
0.0
0.5
1.0
Correlation
CERAD Diffuse Plaque Score & LATE−NC
ROSMAP
CFAS
CC75C
Summary
−0.5
0.0
0.5
1.0
Correlation

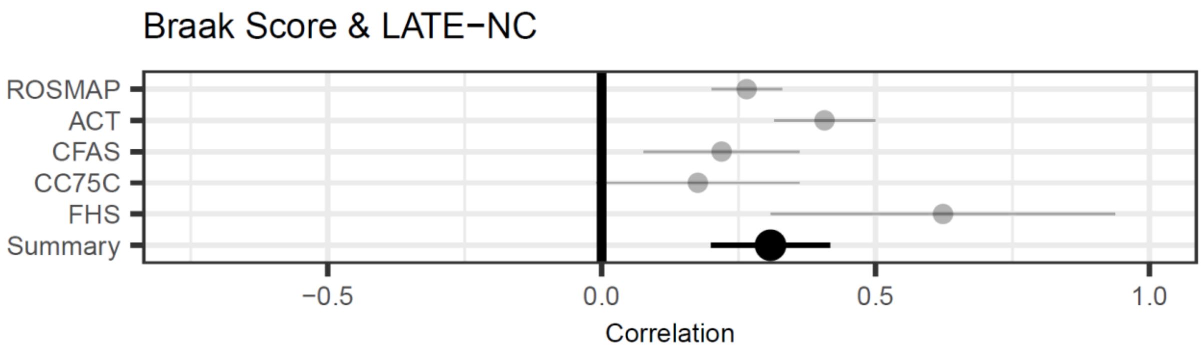
Braak Score & LATE−NC
ROSMAP
ACT
CFAS
CC75C
FHS
Summary
−0.5
0.0
0.5
1.0
Correlation

Appendix Table S2. Numbers of individuals with and without dementia for different combinations of neuropathology co-occurrence

This table shows the data underlying Figure 3 in the main manuscript. Percentage with dementia is calculated excluding individuals with missing dementia status.

Macro-infarcts	Micro-infarcts	High Braak Stage	Lewy Bodies	Severe Neuritic Plaques	LATE-NC	Dementia	Missing	No dementia	% Dementia
						37	13	202	15
					X	39	9	67	37
				X		55	18	149	27
				X	X	45	5	46	49
			X			14	3	34	29
			X		X	12	2	12	50
			X	X		22	7	31	42
			X	X	X	19	2	5	79
		X				8	0	1	89
		X			X	2	2	5	29
		X		X		69	12	35	66
		X		X	X	88	11	22	80
		X	X			1	0	2	33
		X	X		X	3	0	0	100
		X	X	X		33	3	5	87
		X	X	X	X	47	8	4	92
	X					34	6	89	28
	X				X	30	2	19	61
	X			X		21	12	52	29
	X			X	X	21	2	14	60
	X		X			6	2	11	35
	X		X		X	8	0	1	89
	X		X	X		10	1	8	56
	X		X	X	X	9	1	3	75
	X	X				4	1	2	67
	X	X			X	5	0	3	62
	X	X		X		40	4	17	70
	X	X		X	X	55	2	8	87
	X	X	X			1	0	2	33
	X	X	X		X	0	0	1	0
	X	X	X	X		7	1	3	70
	X	X	X	X	X	20	3	3	87
X						24	6	64	27
X					X	19	3	24	44

X				X		34	5	44	44
X				X	X	22	3	14	61
X			X			9	1	17	35
X			X		X	8	0	1	89
X			X	X		16	0	10	62
X			X	X	X	8	3	5	62
X		X				0	0	2	0
X		X			X	1	0	0	100
X		X		X		30	6	12	71
X		X		X	X	43	2	13	77
X		X	X			1	0	0	100
X		X	X		X	1	0	0	100
X		X	X	X		7	1	0	100
X		X	X	X	X	10	1	0	100
X	X					29	6	52	36
X	X				X	17	5	19	47
X	X			X		41	6	43	49
X	X			X	X	27	3	15	64
X	X		X			13	1	13	50
X	X		X		X	4	0	3	57
X	X		X	X		12	1	8	60
X	X		X	X	X	6	0	1	86
X	X	X				3	0	1	75
X	X	X			X	2	0	1	67
X	X	X		X		41	4	16	72
X	X	X		X	X	45	5	3	94
X	X	X	X			1	0	0	100
X	X	X	X		X	0	0	0	NA
X	X	X	X	X		9	1	1	90
X	X	X	X	X	X	12	2	0	100

Appendix Figure S4. Comparison of weighted and unweighted estimates of the distribution of neuropathologic burden in the ACT study

Each panel shows estimates for one of the two different age groups considered across the different neuropathologies included in ACT. Color is used to represent the different neuropathologies and there is one point shown for every category within a specific pathology. For a binary pathology such as macroinfarcts, there are two points: one for the proportion of decedents with macroinfarcts and one for the proportion of decedents without macroinfarcts. In contrast, there are four points shown for CERAD neuritic plaques as there are four categories within this measure. Lines indicate perfect agreement between weighted and unweighted estimates.

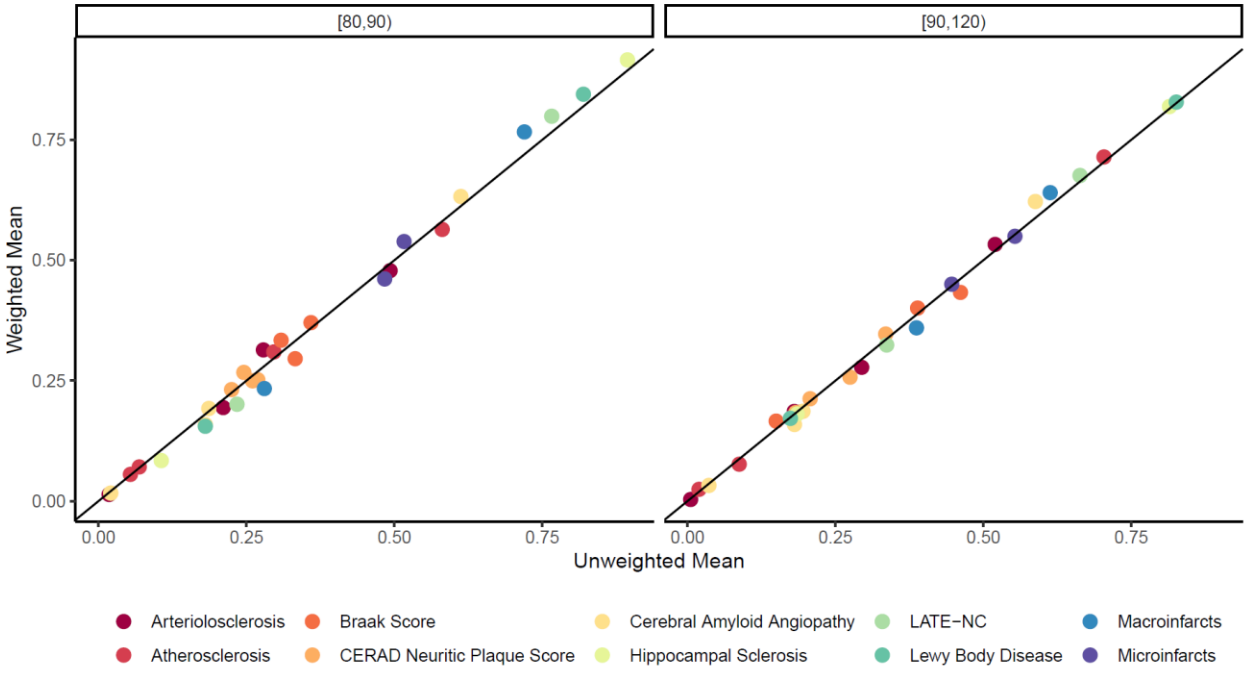

Appendix Figure S5. Comparison of weighted and unweighted estimates of the correlations between neuropathologies in the ACT study

Each point in the plot represents a correlation between two neuropathologies and compares the weighted and unweighted correlation. The line shows perfect agreement between weighted and weighed estimates.

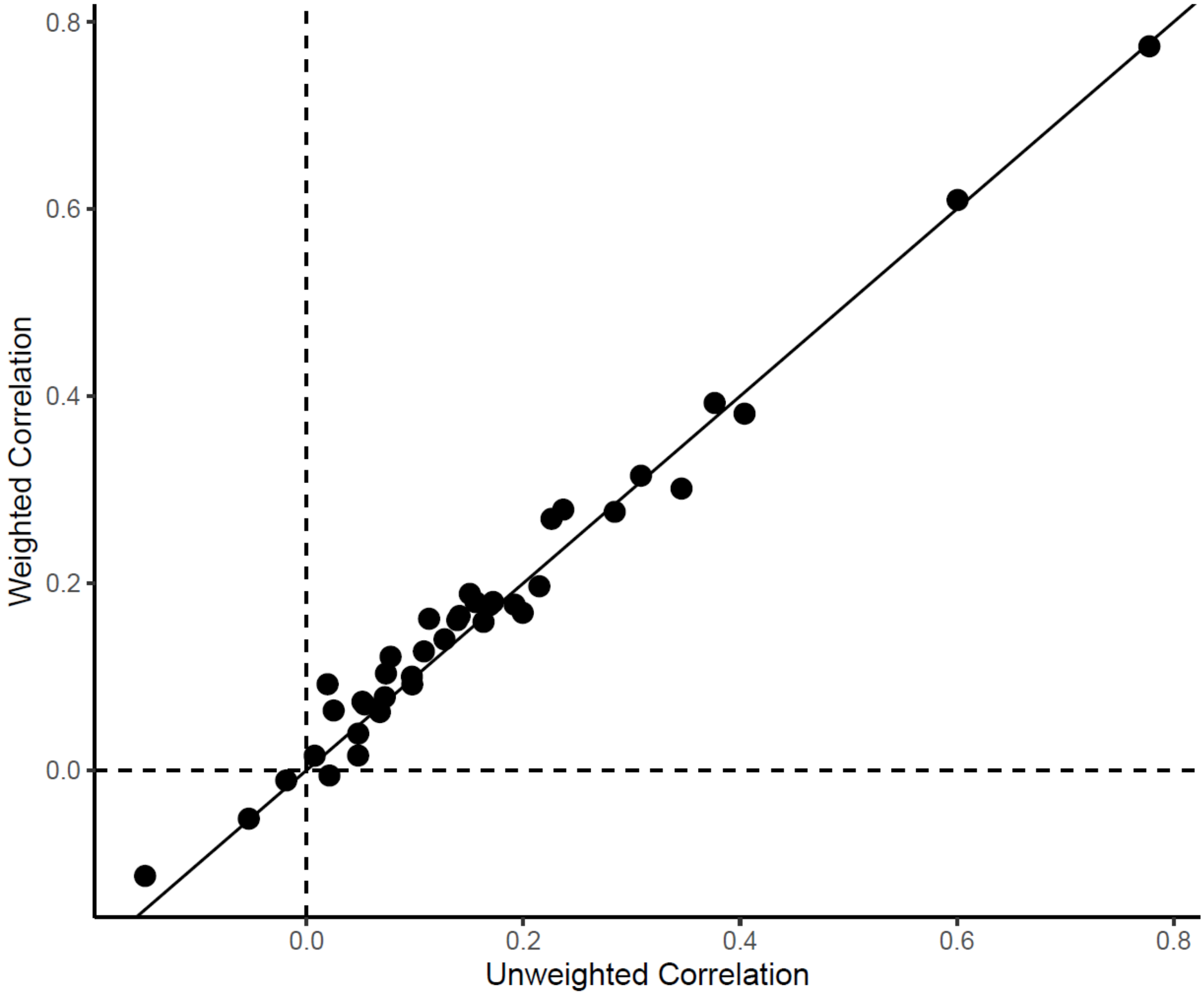

Appendix 5: STROBE Checklist

	Item No	Recommendation	Location
**Title and abstract**	1	(*a*) Indicate the study's design with a commonly used term in the title or the abstract	Title
		(*b*) Provide in the abstract an informative and balanced summary of what was done and what was found	Abstract
**Introduction**			
Background/rationale	2	Explain the scientific background and rationale for the investigation being reported	Introduction (paragraphs 2-3)
Objectives	3	State specific objectives, including any prespecified hypotheses	Introduction (paragraph 4)
**Methods**			
Study design	4	Present key elements of study design early in the paper	Introduction (paragraph 4)
Setting	5	Describe the setting, locations, and relevant dates, including periods of recruitment, exposure, follow-up, and data collection	Methods (paragraph 1); Appendix 1
Participants	6	(*a*) Give the eligibility criteria, and the sources and methods of selection of participants	Methods (paragraph 1)
Variables	7	Clearly define all outcomes, exposures, predictors, potential confounders, and effect modifiers. Give diagnostic criteria, if applicable	Methods (paragraphs 1-2); Appendix 3, Appendix Table S1
Data sources/ measurement	8*	For each variable of interest, give sources of data and details of methods of assessment (measurement). Describe comparability of assessment methods if there is more than one group	Appendix Table S1
Bias	9	Describe any efforts to address potential sources of bias	Methods (paragraph 7)
Study size	10	Explain how the study size was arrived at	Methods (paragraph 1)
Quantitative variables	11	Explain how quantitative variables were handled in the analyses. If applicable, describe which groupings were chosen and why	Methods (paragraph 2); Appendix 3
Statistical methods	12	(*a*) Describe all statistical methods, including those used to control for confounding	Methods (paragraphs 4-6)

		(*b*) Describe any methods used to examine subgroups and interactions	N/A
		(*c*) Explain how missing data were addressed	Methods (paragraph 4); Appendix Fig S1
		(*d*) If applicable, describe analytical methods taking account of sampling strategy	N/A
		(*e*) Describe any sensitivity analyses	Methods (paragraph 7)
**Results**			
Participants	13*	(a) Report numbers of individuals at each stage of study—eg numbers potentially eligible, examined for eligibility, confirmed eligible, included in the study, completing follow-up, and analysed	Methods (paragraph 1)
		(b) Give reasons for non-participation at each stage	N/A
		(c) Consider use of a flow diagram	N/A
Descriptive data	14*	(a) Give characteristics of study participants (eg demographic, clinical, social) and information on exposures and potential confounders	Results (paragraph 1); Table 1
		(b) Indicate number of participants with missing data for each variable of interest	Appendix Fig S1
Outcome data	15*	Report numbers of outcome events or summary measures	Table 1
Main results	16	(*a*) Give unadjusted estimates and, if applicable, confounder-adjusted estimates and their precision (eg, 95% confidence interval). Make clear which confounders were adjusted for and why they were included	Results (paragraph 4-7); Figures 1-3
		(*b*) Report category boundaries when continuous variables were categorized	Appendix 3
		(*c*) If relevant, consider translating estimates of relative risk into absolute risk for a meaningful time period	N/A
Other analyses	17	Report other analyses done—eg analyses of subgroups and interactions, and sensitivity analyses	Results (paragraph 8); Appendix Figs S4-5
**Discussion**			

Key results	18	Summarise key results with reference to study objectives	Discussion (paragraph 1)
Limitations	19	Discuss limitations of the study, taking into account sources of potential bias or imprecision. Discuss both direction and magnitude of any potential bias	Discussion (paragraph 8)
Interpretation	20	Give a cautious overall interpretation of results considering objectives, limitations, multiplicity of analyses, results from similar studies, and other relevant evidence	Discussion (paragraphs 2-6,9)
Generalisability	21	Discuss the generalisability (external validity) of the study results	Discussion (paragraph 8)
**Other information**			
Funding	22	Give the source of funding and the role of the funders for the present study and, if applicable, for the original study on which the present article is based	Funding statement and acknowledgements; Methods (paragraph 8)

*Give information separately for exposed and unexposed groups.

Note: An Explanation and Elaboration article discusses each checklist item and gives methodological background and published examples of transparent reporting. The STROBE checklist is best used in conjunction with this article (freely available on the Web sites of PLoS Medicine at http://www.plosmedicine.org/, Annals of Internal Medicine at http://www.annals.org/, and Epidemiology at http://www.epidem.com/). Information on the STROBE Initiative is available at www.strobe-statement.org.
